# Unraveling Polymorphic Control in the Solid-State [2 + 2] Cycloaddition of Vitamin K_3_: Insights from Single-Crystal Irradiation

**DOI:** 10.1021/jacs.5c06303

**Published:** 2025-06-04

**Authors:** Fabio Loprete, Lorenzo Pandolfi, Andrea Giunchi, Sara Pandolfi, Simone d’Agostino, Riccardo Tarroni, Patrizio Graziosi, Raffaele G. Della Valle, Tommaso Salzillo, Elisabetta Venuti

**Affiliations:** † Dipartimento di Chimica Industriale “Toso Montanari”, Università di Bologna, Via Piero Gobetti 85, 40129 Bologna, Italy; ‡ Dipartimento di Chimica “Giacomo Ciamician”, Università di Bologna, Via F. Selmi 2, 40126 Bologna, Italy; § CNR - Institute for Nanostructured Materials (ISMN), Via Piero Gobetti 101, 40129 Bologna, Italy

## Abstract

The solid-state [2 + 2] cycloaddition of vitamin K_3_ (VK3) polymorphs has been systematically reexamined, overcoming the limitations of powder-based studies by focusing on single-crystal behavior. Using THz micro-Raman and FTIR-ATR vibrational spectroscopies combined with X-ray diffraction and solid-state density functional theory (DFT) calculations, we unveil distinct photoreactivity and stereoselectivity in both polymorphs. Monochromatic irradiation at the absorption tail preserved the crystal integrity, enabling a detailed investigation of the reaction outcomes. While both polymorphs undergo photoreaction, their transformations proceed via distinct crystal-to-crystal processes, yielding *cis-syn* and *cis-anti* dimers selectively. The photochemical pathway to the formation of the *cis*-isomers is analyzed by quantum mechanical calculations. These findings highlight the critical role of polymorphism in solid-state photochemistry while questioning the conventional dichotomy between defect-driven and purely topochemical mechanisms.

## Graphical abstract

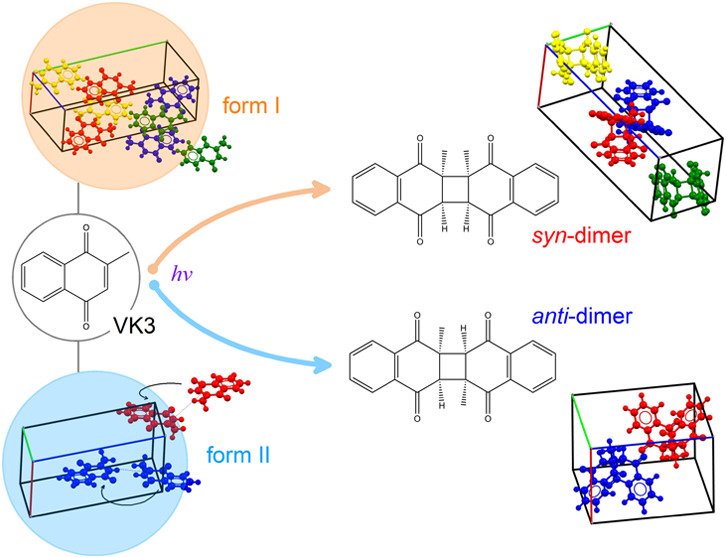

## Introduction

Menadione (2-methyl-1,4-naphthoquinone), also known as vitamin K_3_ (VK3), is widely used as a nutritional supplement in animal feed and, more importantly, as an intermediate in the synthesis of vitamin K.^1^ However, its low solubility in water and high sensitivity to light remain significant challenges for its usage and storage, as the compound belongs to the group of molecules that undergo a [2 + 2] dimerization in the solid state.^2–5^ The reaction, initiated by UV light absorption, results in the formation of a dimer characterized by a four-membered ring. Of the four possible dimer isomers illustrated in Scheme 1, previous studies report that broadband irradiation of the crystalline powder of the commercial product, composed of the crystal form hereafter indicated as VK3-I, yields only two isomers in equal amounts: namely, the *cis-syn* and the *cis-anti* dimers, as shown in Figure S1.^2,3^

**Scheme 1:**
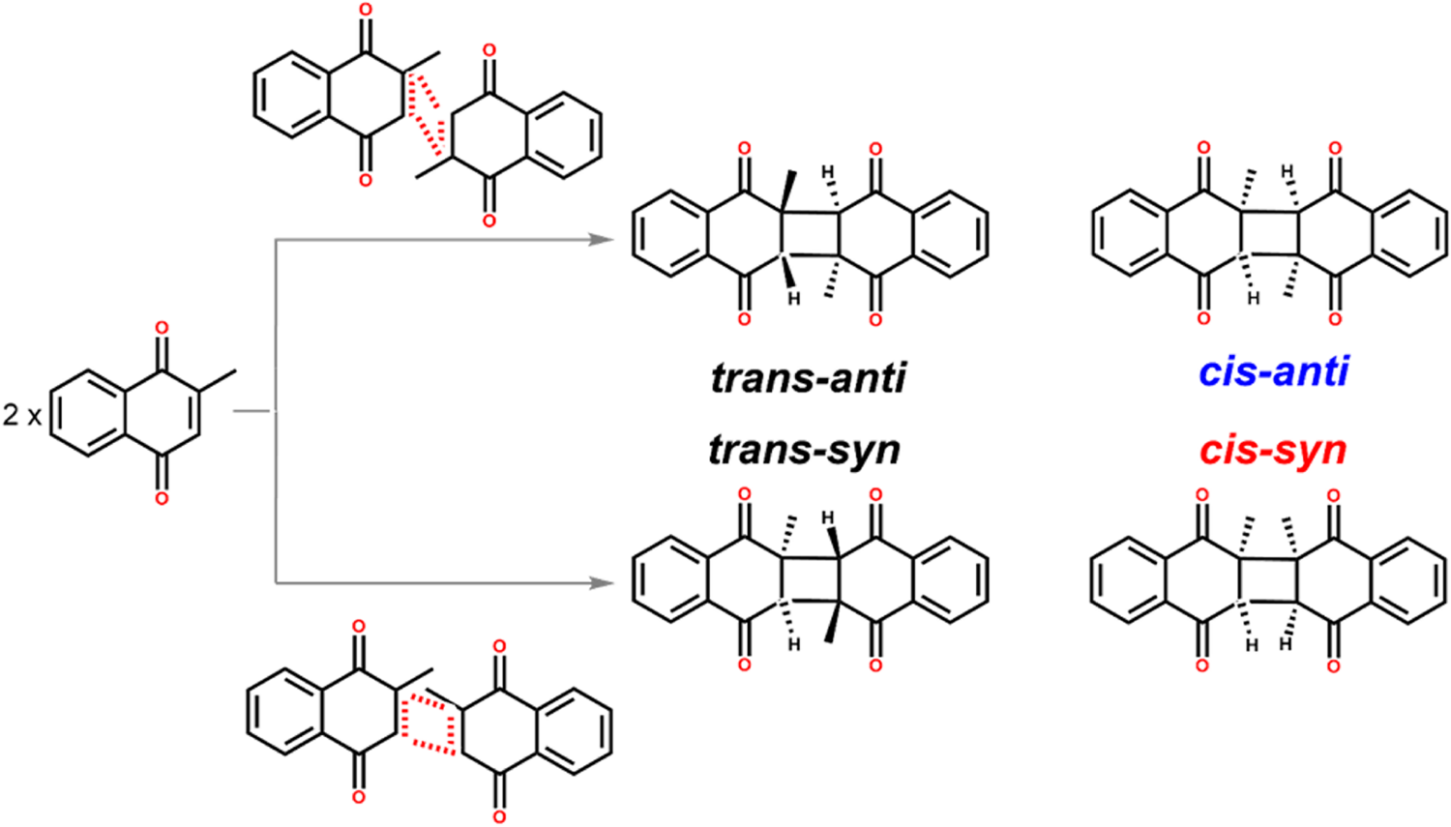
Photodimerization Reaction Pathways of VK3, Displaying the Four Expected Products^a^

a Only the *cis-syn* and *cis-anti* dimers are reported as being obtained in the solid state.^3^

The photochemical reactivity of VK3 has indeed been the subject of investigation for decades. In 1956, Asahi examined it in detail in both solution and solid state,^2^ reporting the formation of the epoxy-1,4-naphthoquinone in solution and providing the first IR reference spectra for the dimers produced by crystal irradiation. In 1993, Taira et al. solved the dimers’ crystal structures,^3^ identifying them as *cis*-isomers, but could not determine the structure of the monomer itself, as it was found to decompose under the X-rays. The structure of VK3-I therefore remained unknown until reported by Nowell and Attfield in 2004,^6^ and again in 2008,^7^ along with that of a second polymorph, hereafter called VK3-II. Notably, VK3-I was also identified as the kinetically favored form and VK3-II as the thermodynamically stable one. Due to their distinct packing characteristics, VK3-I and VK3-II exhibit markedly different patterns of intermolecular interactions involving the benzene and quinone rings of the molecule, as described in detail in ref 7 and in Figure S1. These differences suggest that the two forms may also exhibit different photoreaction behaviors. As yet, the photochemical behavior of this system has been considered as an exception^8^ to the topochemical postulate formulated in the pioneering work of Schmidt and Cohen,^9^ which states that cycloadditions in crystals can only occur if reciprocal orientations and distances of the reactive C=C double bonds obey certain empirical constraints. However, exceptions to this postulate are several.^10^ While the postulate effectively highlights the significant impact of molecular topology on crystal reactivity, it assumes a static lattice, where only minimal atomic movements are allowed. In reality, within the solid, substantial molecular motions and significant material transport can occur.^11–13^ Moreover, the role of defects must be considered, as variations in their density and nature from crystal to crystal can significantly alter the geometry and energy of the reacting excited state.^14^

The presence of multiple polymorphs in a reactive system offers a unique opportunity to explore how the product structure depends on packing characteristics. The dimerization of the various crystal forms of *trans*-cinnamic acid has provided an exemplary evidence to the support of the topochemical hypothesis.^9,15,16^ In the case of VK3, the occurrence of two known polymorphs enables an analysis of this relationship under conditions where the strict criteria for correlating product geometry with the reactant lattice are less clearly satisfied. In addition, investigations have been carried out to date on crystalline powders, which typically exhibit a high concentration of defects, and this has added a layer of complexity to the previous analyses.

This work aims to provide a fresh perspective on the solid-state photoreactivity of VK3 by focusing on the behavior of single crystals of its two polymorphs rather than limiting the analysis to crystalline powders. The study employs micro-Raman and FTIR-ATR spectroscopies, techniques that have previously been demonstrated to be effective in monitoring photochemical reactions.^17–19^ The approach involves tracking the compound’s photochemical transformation and its kinetics by detecting its intramolecular vibrations, while changes in the structure are identified by examining the evolution of the low-energy lattice-phonon pattern.

Upon irradiation, the single crystals of both polymorphs were found to exhibit photoreactivity. Moreover, previously unreported stereoselectivity in the formation of the *cis-*isomers was detected, depending on the specific crystal form.

## Experimental Methods

### Crystal Growth

Crystals of VK3-I (Sigma-Aldrich) and VK3-II were obtained through slow solvent evaporation from a methanol solution at 300 and at 277 K, respectively.^7^ VK3-I grew as needle-shaped crystals, and VK3-II crystallized as prismatic crystals. The different temperatures at which the solvent is left to evaporate appear to be the only factor driving the growth toward either form, as crystallization from acetone was also successful in yielding both.

### Solid-State UV Irradiation

The single-crystal samples were irradiated with LED sources (λ = 405 ± 5 and 530 ± 5 nm) placed at 1.5 cm. The same 405 nm light source and distance were also used to irradiate the powders for FTIR-ATR and micro-Raman measurements. The powders were prepared by finely grinding the commercial crystalline material in a mortar. Approximately 1 mg of this was irradiated and continuously mixed during the process to ensure the exposure of fresh materials and prevent surface phenomena. FTIR-ATR spectra of the powder were recorded at intervals of 10–30 min until the spectrum either coincided with that of the pure dimer or showed no further change.

### Single-Crystal X-ray Diffraction

Single-crystal data for VK3-I and VK3-II were collected at 297 and 200 K, respectively, on an Oxford XCalibur S CCD diffractometer equipped with a graphite monochromator (Mo–Kα radiation, λ = 0.71073 Å) and with a cryostat Oxford CryoStream800. Crystals of VK3-I showed twinning, and the reflection data were integrated with the default configuration for twinned crystals of the CrysAlisPro software. The structures were solved by intrinsic phasing with SHELXT^20^ and refined on F^2^ by full-matrix least-squares refinement with SHELXL^20^ implemented in the Olex2 software.^21^ H_CH_ atoms were added in calculated positions and refined on their respective carbon atoms. Crystal data can be obtained free of charge via www.ccdc.cam.ac.uk/conts/retrieving.html (or from the Cambridge Crystallographic Data Centre, 12 Union Road, Cambridge CB21EZ, UK; fax: (+44)­1223-336-033; or email: deposit@ccdc.cam.ac.uk); CCDC numbers 2441957–2441959.

### ATR Spectroscopy

FTIR-ATR measurements in the wavenumber range 400–4000 cm^–1^ with a resolution of 2 cm^–1^ were carried out on a PerkinElmer Spectrum Two spectrophotometer equipped with a Universal ATR accessory, which features a single-reflection diamond crystal. Typically, 16–64 coadded scans per spectrum were collected, depending on the sample characteristics and signal-to-noise ratio.

### HPLC

Semi-preparative HPLC was performed using a Phenomenex Luna C18(2) column (6 × 150 mm^2^, 5 μm, 100 Å) with a reversed-phase stationary phase. The system, controlled by a Waters 600 pump, used a gradient elution of water and acetonitrile, optimized for dimers’ separation.

### Raman Spectroscopy

Raman spectra were recorded with a Horiba Jobin Yvon T64000 spectrometer equipped with three monochromators in a double subtractive configuration. The spectrometer was coupled to an Olympus BX40 confocal microscope equipped with 100, 50, 20, and 10× objectives, for a lateral resolution lower than 1 μm with the 100× objective. This allows gathering information about the composition in crystal domains of micrometric dimensions. The excitation wavelength used was the 647.1 nm line from a tunable Kr^+^ gas laser, with a nominal power of 1 W. The power was reduced by neutral density filters to avoid sample damage.

Crystallographic data of the VK3 monomers and dimers. The literature crystallographic data^7^ for the two known polymorphs VK3-I and VK3-II are reported in Table S1. The data from single crystals of this work, obtained from samples with needle and prismatic morphologies as described in the literature (Figure 1), are provided in Table S2.

**Figure 1:**
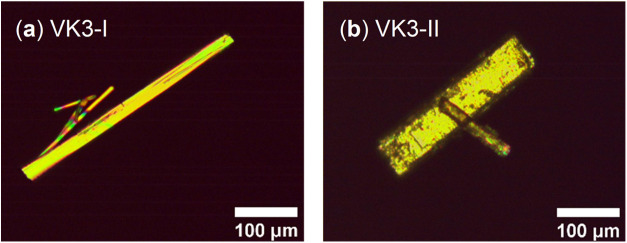
Cross-polarized optical image of the single crystals of (a) polymorph VK3-I and (b) polymorph VK3-II.

Both VK3 monomeric structures given in ref 7 belong to the monoclinic *P*2_1_/*c* space group. However, the *P*2_1_/*c* structure of the VK3-II polymorph corresponds to the *P*2_1_/*n* structure determined in this work once reduced to its Niggli cell, as shown in Table S1. VK3-I contains *Z* = 8 molecules per cell, with the asymmetric unit comprising two nonequivalent molecules, while VK3-II contains *Z* = 4 molecules per cell, with the asymmetric unit comprising one molecule. VK3-I and VK3-II both display intermolecular C–H···O interactions involving the same atoms, but they connect molecules in different ways.^7^ As shown in Figure S1, in VK3-I, three such contacts link two nonequivalent molecules into 2D layers in the *bc* plane. VK3-II, instead, forms similar layers through two weaker C–H···O bonds between symmetry-related molecules. These differences affect the overall packing: VK3-I has nonplanar layers formed by its asymmetric unit, while VK3-II forms flat layers through translational symmetry. Between layers, VK3-I shows little aromatic stacking, whereas VK3-II features strong π–π overlap between quinonoidal and aromatic rings at a distance of 3.55 Å.

The selective formation of the *cis*-dimers as the predominant photoproducts has been extensively confirmed^3–5^ and attributed to the reciprocal orientation of molecules in the crystal state. Indeed, the formation of the equally energetically favored *trans*-dimers, which possess lower steric hindrance, would be expected.

The crystallographic data for two VK3 photoproducts^3^ are given in Table S3. These structures correspond to the *cis-anti* and *cis-syn* VK3 dimers, the isomers reported to be obtained by photoreaction in the solid state in an approximately 1:1 ratio. The literature structures were obtained following recrystallization from benzene of powders irradiated with black light.^3^ The *cis-syn* dimer of VK3 crystallizes in a monoclinic *C*2/*c* lattice, with *Z* = 8 molecules per cell, while the *cis-anti* dimer was found to adopt a triclinic *P*1̅ unit cell with *Z* = 2 molecules. Table S3 also reports our structural redetermination for the *cys-anti* dimer, which revealed a higher symmetry corresponding to *C*2/*c*. However, the literature *P*1̅ cell closely matches the Niggli-reduced cell of our structure, showing that the two structures are equivalent. Despite slight differences in molecular geometry, the *anti*-dimer is recognized as the energetically favored species (see the SI). Notably, the *syn*-dimer structure has a lower density and consequently a melting point lower than that of the *anti*-dimer. It should also be noted that the literature *anti*-dimer structure is a racemic mixture of two enantiomers, deriving from the two possible molecular geometry reactions, where bonds are formed either above or below the molecular planes.

## Computational Methods

Density functional theory (DFT) calculations were performed using the Vienna Ab initio Simulation Package (VASP 6.3)^22,23^ to investigate the Raman spectra of the VK3-I and VK3-II polymorphs and of the *cis*-dimers with a focus on the lattice-phonon energy range for polymorph identification.

We employed the Perdew–Burke–Ernzerhof (PBE) pseudopotentials^24^ and a posteriori van der Waals (VdW) corrections using the D3-BJ Grimme method with Becke–Johnson damping functions^25^ to account for long-range dispersion interactions. Calculations started from the experimental unit cell parameters and atomic positions. The k-point grid and energy cutoff were optimized for efficiency and accuracy, starting with a 400 eV cutoff and a convergence criterion of 1 × 10^–8^. The final k-point samplings and energy cutoffs were 6 × 2 × 4 (1000 eV) for VK3-I, 5 × 4 × 2 (900 eV) for VK3-II, 6 × 6 × 2 (1000 eV) for *cis-syn*, and 5 × 5 × 3 (900 eV) for *cis-anti*. Atomic positions were relaxed with increasing force convergence criteria (10^–4^–10^–6^), iterating until no further optimization was possible. At the final step, the wave function convergence criterion was tightened to 10^–9^. The obtained structures were used to calculate the vibrational modes. In this step, the Phonopy package was utilized,^26,27^ with a 2 × 2 × 2 supercell and the energy cutoffs defined above. The Raman spectra intensities were obtained using the vasp_raman.py script^28^ and finally adjusted for excitation wavelength and temperature effects to match experimental conditions.^29^

For the excited state electronic calculations, an isolated molecule approach was adopted. Optimization of the equilibrium geometries of the monomer and four dimers were carried out by means of the ORCA 4.2.1 suite of quantum chemistry programs.^30,31^ The geometries were optimized in vacuo using the B3LYP functional^32^ and the def2-TZVP basis.^33^ The absorption spectrum of the monomer was calculated by means of the TDDFT method,^34^ using the same functional and basis set. TDDFT was then used to identify geometries and energies of the S_0_/S_1_ conical intersection, i.e., the minimum-energy crossing point (MECI).^35,36^ TDDFT results on conical intersections were checked using the Complete Active Space Self-Consistent Field method (CASSCF), as implemented into the Molpro program.^37^ DFT simulations were also used to compute Raman and IR spectra of both the monomer- and the dimer-isolated molecules. The calculated wavenumbers were scaled for a factor of 0.967, suited for this combination of exchange correlation functional and basis set.^38^ The Raman intensities were adjusted for the excitation wavelength and temperature effects.

## Results

### Spectroscopic Characterization of the VK3 Polymorphs

The low-frequency region of the Raman spectrum, where lattice-phonon modes arising from intermolecular interactions appear, is shown in Figure 2a for both VK3-I and VK3-II. The two polymorphs exhibit distinct spectral patterns due to differences in molecular packing and unit cell composition. Comparison with the commercial powder spectrum confirms that it consists entirely of the VK3-I form. DFT simulations of the spectra are provided in Figure S2, with calculated wavenumbers listed in Table S6. There is excellent agreement between the experimental and calculated wavenumbers for both polymorphs, and the less satisfactory agreement of the band relative intensities, particularly for the VK3-I form, must be ascribed to polarization effects inherent in the experimental crystal spectra. The lattice-phonon character of a computed mode can be determined in the analysis of the DFT vibrational results via the (squared) projections of the mode eigenvector around and along the three molecular axes of inertia, and the results are also given in Table S6. Due to the molecular rigidity of vitamin K_3_, as many as 21 modes within the 0–100 cm^–1^ range in VK3-I and 10 in VK3-II exhibit a predominantly translational and rotational character, involving the motion of the molecule as a whole. From a dynamic perspective, lattice phonons can play a crucial role in facilitating solid-state reactions. These vibrational motions are analogous to molecular collisions in the gas phase, where intermolecular interactions promote molecular rearrangements and activate reaction pathways. Consequently, phonon-driven mechanisms can significantly influence the reactivity of materials in the solid state.

**Figure 2:**
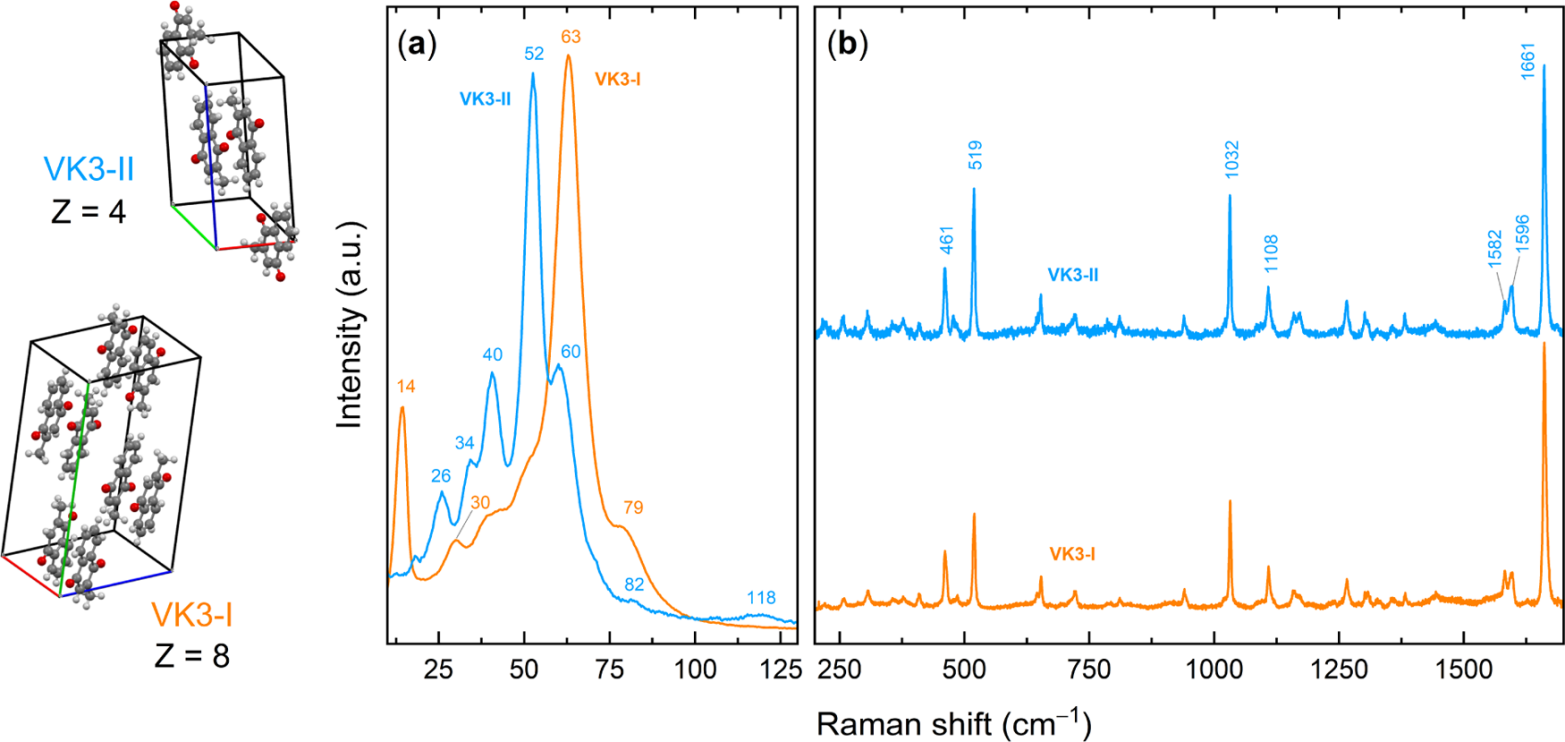
Raman spectra of VK3-I and VK3-II polymorphs: (a) low wavenumber region of the lattice phonons; (b) 200–1800 cm^–1^ range of the intramolecular vibrations.

The Raman spectra of the polymorphs in the wavenumber range of 200–1700 cm^–1^ are reported in Figure 2b. In the high-wavenumber region, where the bands arise from intramolecular modes, the spectral signatures of the polymorphs are remarkably similar. This behavior aligns with the nature of packing polymorphism, which typically does not involve significant conformational changes, particularly for a rigid molecule like vitamin K_3_. The crystal packing exerts only a minor perturbation on the molecular force field, resulting in mode frequencies that closely mirror those observed in the gas phase. The experimental spectra have been accurately assigned using DFT simulations of the Raman spectrum both for the isolated molecule and the crystal, yielding excellent agreement with the observed data.

The crystallographic center of symmetry in both VK3-I and VK3-II dictates distinct Raman and IR spectral features, with only gerade modes appearing in Raman spectra and ungerade modes in IR spectra. To access these ungerade vibrations, FTIR spectra were recorded (Figure 3). While the molecular vibration range of both polymorphs exhibits similar spectral characteristics, minor differences arise due to the distinct number of molecules per unit cell, aiding polymorph discrimination and are indicated in Figure 3. This applies to the C–H stretching range above 2900 cm^–1^, and in the fingerprint region of the in plane C–H bending modes, where VK3-I presents a characteristic doublet at 901 and 885 cm^–1^, while VK3-II displays a single band in this region. Comparison with DFT-simulated IR spectra enabled the assignment of key vibrational modes, including the C=O at 1659 cm^–1^ and the C=C stretching at 1622 cm^–1^. Beyond structural insights, these assignments are instrumental in determining reaction kinetics, as will be discussed later (see also the SI for a more detailed assignment of the most intense bands in this region).

**Figure 3:**
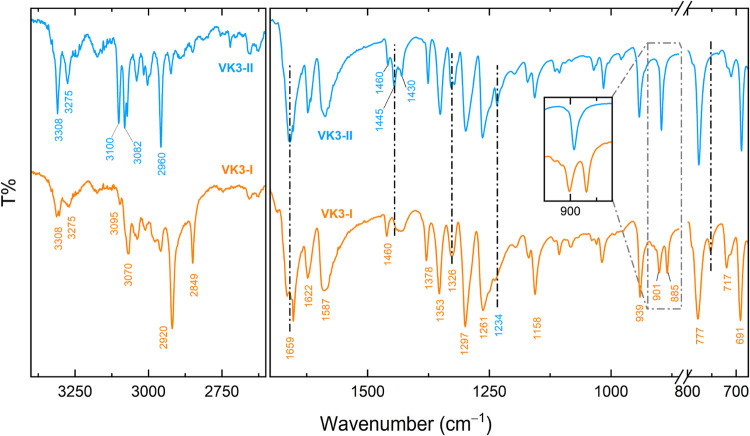
FTIR spectra of the VK3 polymorphs.

### Dimerization Photoreactions of Single Crystals

The investigation of the reactivity of VK3 single crystals focused on the analysis of lattice phonons, which serve as markers of lattice transformation. In addition, the evolution of the phonons over time can provide crucial insights into the nature of the reaction, distinguishing between homogeneous and heterogeneous processes.^39,40^ The separate inspection of the two monomer polymorphs enabled us to investigate whether their different packing would result in a preferential reaction path toward one of the possible dimerization products. This approach is in fact critical in understanding the polymorph-dependent reactivity and possibly the role of phonon dynamics in the solid-state reaction.

A commonly adopted strategy in irradiating crystalline samples^15^ is to excite in the red tail of the spectrum, i.e., where the absorption is lower. Under these conditions, light penetration becomes more uniform throughout the crystal, ensuring that photoproduct formation is not limited to the surface but instead is evenly distributed. This promotes the formation of a solid solution and facilitates a seamless crystal-to-crystal phase transition. The UV–Vis absorption spectrum of VK3, which guides the selection of the irradiation wavelength, is given in Figure S3 (top), and shows a maximum near 300 nm, with a long tail extending to longer wavelengths. The analysis of the TDDFT results reported in the same Figure S3 is crucial for understanding the electronic states involved in the absorption process and subsequently involved in the reaction pathways. The two lowest absorptions, at wavelengths >400 nm and with negligible oscillator strength, involve transitions from the HOMO and HOMO–1 orbitals,^41^ which are basically n-type orbitals located on the oxygen atoms, to the LUMO π* orbital located on the quinoid ring. Two other transitions follow, at wavelengths between 300 and 350 nm and with medium oscillator strength. These involve the nearly degenerated HOMO–2 and HOMO–3 π orbitals, located in the benzenoid ring and the LUMO π*. The most intense absorption below 250 nm involves a transition from the HOMO–4 π orbital, located at the quinoid ring, to the LUMO π*. After accounting for the bathochromic shift of the absorption in the solid state, particularly affecting the π- π* portion of the spectrum, this shows that the weak *S*_0_ → *S*_1_, *S*_2_ transitions fall within the experimental long-wavelength tail. Thus, irradiation in this region meets the requirement of achieving uniform light penetration and an even photoproduct distribution. Accordingly, monochromatic irradiation at 405 nm was chosen.

The effect of 405 nm irradiation on a single crystal of VK3-I is shown in Figure 4a. The sample does not undergo any shattering, cracking, or discoloring. While its morphology is maintained polarized, light images reveal a progressive loss of its single-crystal nature starting from the edges: the needle in the figure, initially oriented along the direction of maximum intensity under crossed polarization, gradually darkens over time. The brown discoloring observed in the powder of the VK3-I phase subjected to intense UV light (see Figure S4) demonstrates that side reactions may occur. However, with single crystals, the formation of undesired products can be controlled and kept to a minimum with the excitation in the tail of the absorption spectrum and low light intensity.

**Figure 4:**
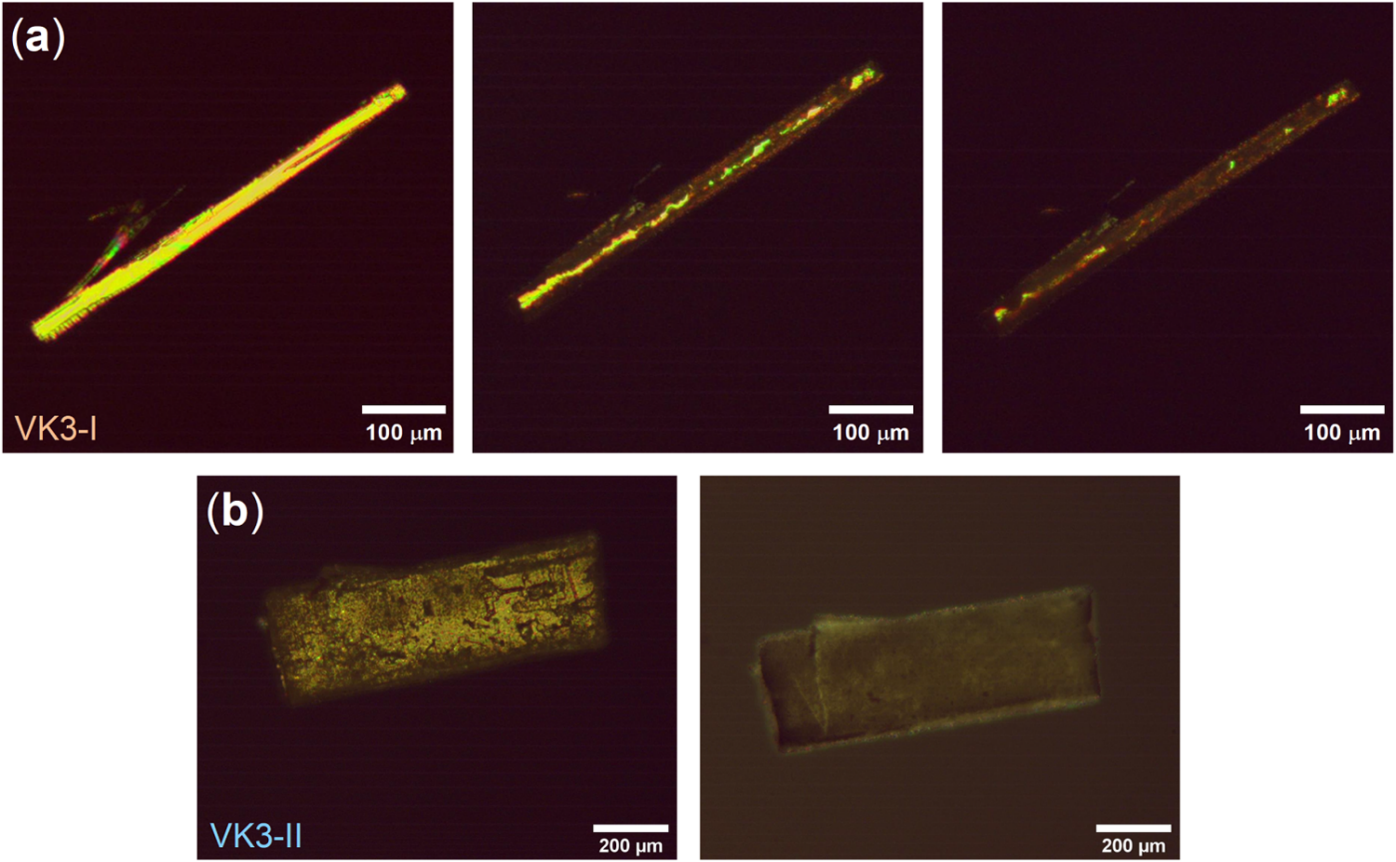
Cross-polarized optical images of (a) VK3-I and (b) VK3-II single crystals during the photoirradiation process.

Upon irradiation, a distinct different pattern emerges in the Raman lattice-phonon spectra of VK3-I (Figure 5a, top). At shorter irradiation times, this pattern coexists with that of the reactant, indicating the simultaneous presence of both the monomer and the dimer photoproduct, thereby demonstrating a crystal-to-crystal transformation of a heterogeneous nature. The detection of both monomer and dimer lattice phonons in the Raman measurements at a certain stage indicates that the solid solution of reactant and product, which must be present at the onset of the reaction,^39^ has vanished. This suggests that the dimer lattice has nucleated and grown within the monomer’s structure. In the high-frequency region of Figure 5b, which shows the spectra of the monomer and photoproduct at the longest irradiation time, spectral differences become evident, revealing chemical changes. The evidence of the dimerization reaction is observed in the 1600–1700 cm^–1^ range, where the C=O stretching band shifts to higher frequencies, indicating the loss of conjugation due to the formation of the four-membered ring. The corresponding disappearance of the C=C stretching band of the monomer is not easily detectable in the Raman spectrum due to its low intensity.

**Figure 5:**
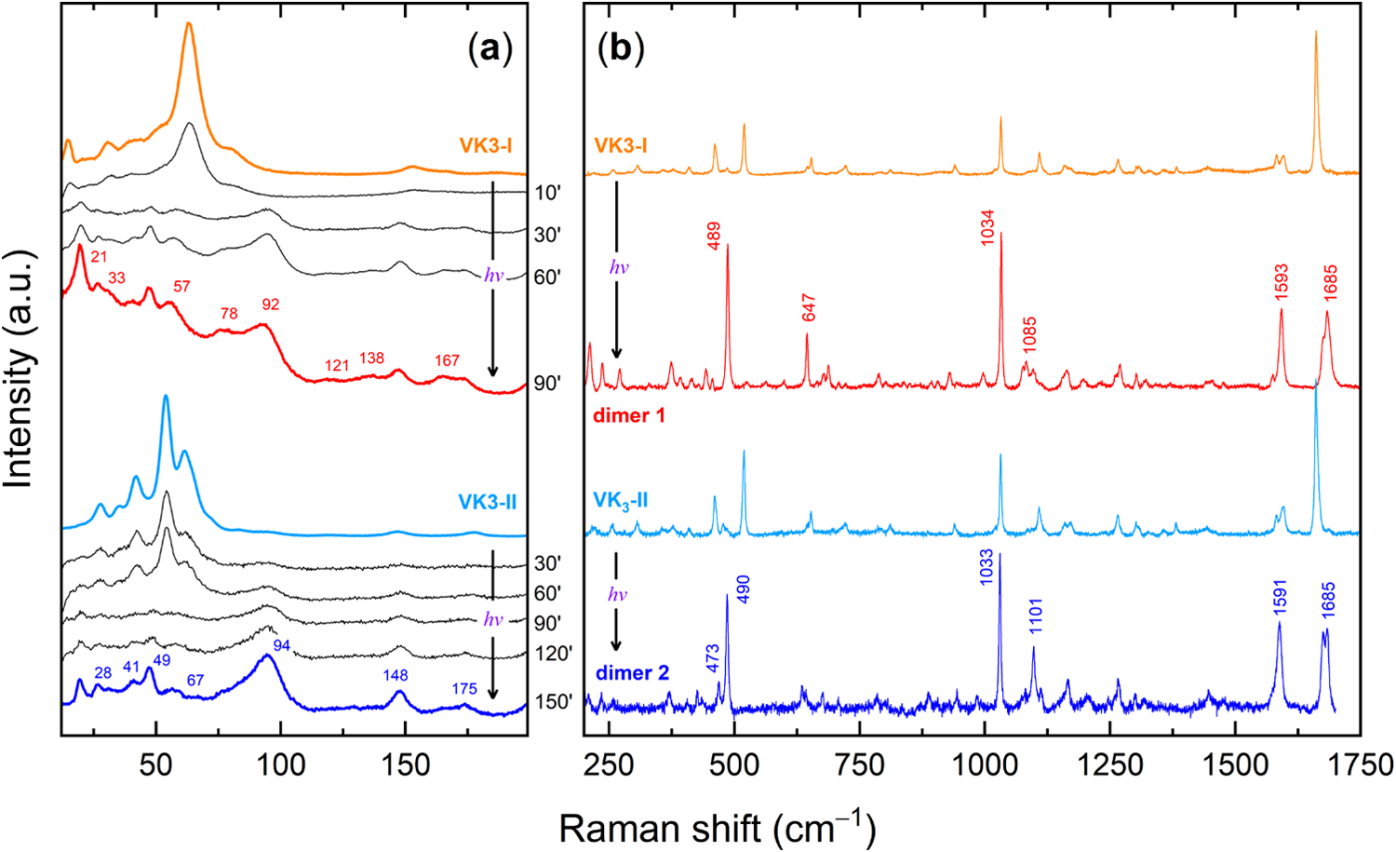
(a) Raman spectra of VK3-I and VK3-II single-crystal polymorphs in the lattice-phonon region and their evolution under a 405 nm irradiation. (b) Raman spectra recorded in the intramolecular vibration range at the beginning and final stages of the same irradiation. The red and blue spectra are assigned to two distinct dimeric products, based on the results obtained from the irradiation of the powder sample (vide infra).

The behavior exhibited by VK3-II under irradiation is also shown in Figure 5. Here too, the lattice-phonon pattern changes (Figure 5a, bottom) but on a slower time scale, with a new spectrum emerging superimposed on that of the monomer. In the region of intramolecular vibrations (Figure 5b) modifications similar to those already observed in the reaction of the VK3-I polymorph are detectable, such as the shift to higher frequencies of the C=O stretching. This result demonstrates that also this polymorph is photoreactive, despite not meeting Schmidt’s topochemical criteria. In fact, while VK3-II exhibits stronger π–π stacking, this occurs between the quinonoidal and aromatic rings of the molecule pairs,^7^ without aligning the reactive double bonds in the favorable orientation for the photodimerization to proceed. Instead, VK3-I, owing to its less tightly packed, nonplanar layers,^7^ might occasionally allow more favorable approaches (see also Figure S1).

Clearly, the spectra of the two photoproducts are not exactly coincident, with the most significant spectral difference found in the lattice-phonon pattern and thus indicating the formation of a different crystal structure. These findings are also noticeable in the set of spectra of Figure S5, analogous to those in Figure 5, but obtained when the two crystalline forms were irradiated at 530 nm. At this wavelength, the samples are nearly transparent, and still the reaction occurs with longer times, allowing for the slower reactivity of VK3-II to be recorded, with a process spanning a period of several hours. The spectral windows of lattice phonons and intramolecular vibrations shown in Figure S5 were recorded simultaneously, allowing direct correlation between chemical and physical transformations. The evolution of the lattice spectrum over irradiation time closely mirrors changes in the molecular vibrations, demonstrating that both processes occur on the same time scale.

Thus, the irradiation of single crystals confirms the reactivity of the VK3-I polymorph, which leads to a photoproduct that remains crystalline, indicating a crystal-to-crystal transformation. Likewise, VK3-II undergoes a similar transformation but yields a distinct crystalline form, different from that of VK3-I. Whether this distinction arises from a different crystal structure of the same chemical species or an entirely different product remains to be determined. Addressing this question required extending the investigation to irradiation of the crystalline powder.

### Photoreaction of the Irradiated Powder

The irradiation of VK3-I powder samples was carried out with a 2-fold objective: first, to reproduce and validate previous literature results, and second, to characterize the dimeric products to support the analysis of the irradiated single crystals. This approach provided a sufficient photoproduct for chemical and spectroscopic characterizations. Among the techniques employed, FTIR spectroscopy proved more effective during powder irradiation, as its greater sensitivity to intramolecular vibration changes allowed for fully tracking the time evolution of the reaction. Conversely, lattice Raman spectroscopy was crucial for determining the crystalline form of the photoproduct, providing essential structural insights.

The progression of the reaction by FTIR was monitored by selecting the bands typically associated with [2 + 2] photoadditions, which served as markers. As mentioned above, these include the disappearing C=C stretching band of the reacting bonds, which gets replaced by the modes of the four-carbon atom cycle, along with the C=O stretching band, which shifts to higher frequencies due to the conjugation loss, as confirmed by the simulations of the spectra in Figure S6. The evolution of the FTIR spectra of the VK3-I powder over the course of irradiation is illustrated in Figure 6, with the region where major changes, derived from the marker bands, are detected. Over the 150 min irradiation time, when not stirred, the VK3 yellow powder turned to a brownish hue on the surface while maintaining its yellow coloration underneath. Given that the dimeric species are not expected to absorb in the visible range, this suggests the formation of additional products.

**Figure 6:**
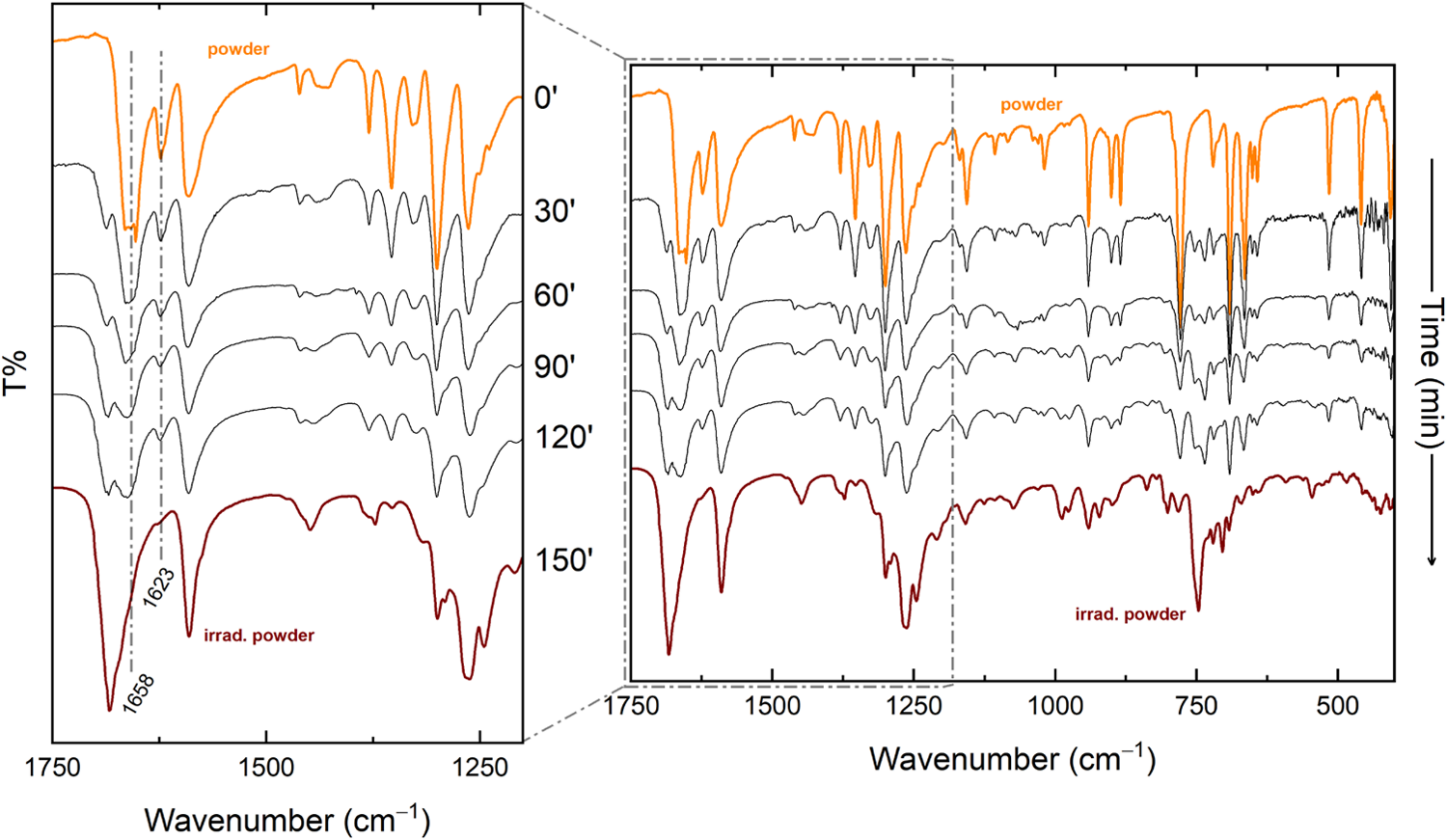
Evolution of the FTIR spectra of VK3-I with irradiation at λ = 405 nm, with a sampling interval of 30 min.

The NMR spectrum of the irradiated powder sample is shown in Figure 7. A small amount of unreacted monomer (orange trace) is still present, as indicated by the chemical shift at 2.17 ppm. The shifts observed at 3.81 and 3.45 ppm (brown spectra) are attributed to the hydrogens of the newly formed four-atom ring, confirming the presence of both *cis-syn* and *cis-anti* photodimers, labeled **B**, **b** and **C**, **c**, respectively, in Figure 7. Based on these shifts, the relative abundance of the dimers is estimated to be 47:53 (see the inset), consistent with literature reports of a 50:50 formation ratio of the *cis*-dimers.^2,3^ Figure S7 compares the experimental powder XRD pattern of the dimer mixture with simulated patterns based on published dimer structures,^3^ showing that the sample is a mixture of the known forms. Following the procedure reported in the literature,^5^ the reacted powder was treated by semi-preparative HPLC yielding the two pure dimers, which were finally isolated as white microcrystals. The quantities were sufficient for recrystallization and growth of a single crystal suitable for XRD analysis of the *cis-anti* dimer only, but they were perfectly adequate for micro-Raman and FTIR spectroscopic characterizations.

**Figure 7:**
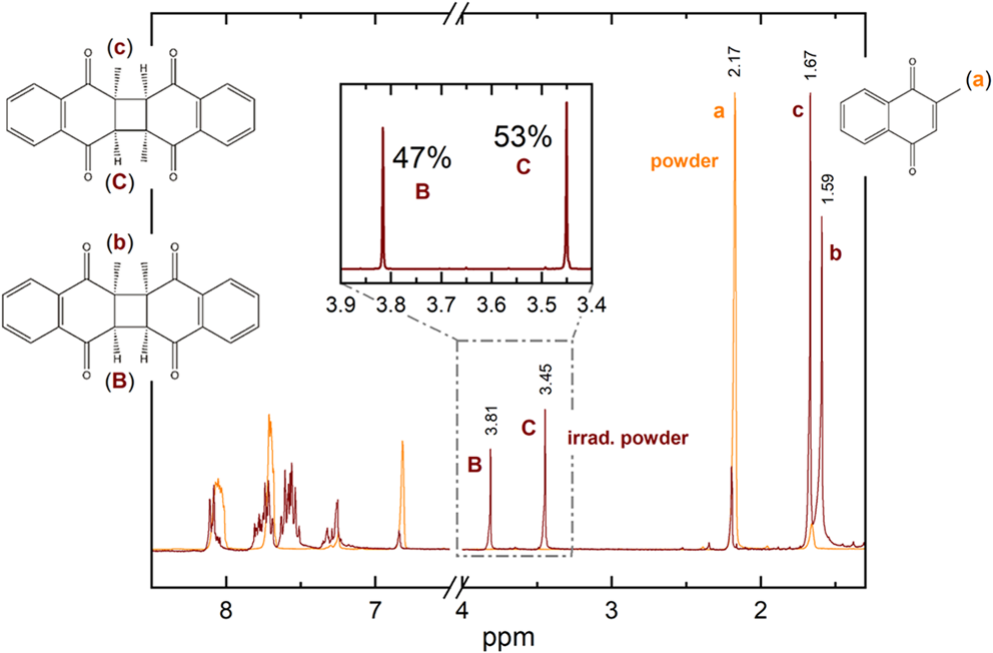
NMR spectra of the commercial powder of VK3 before and after irradiation.

The lattice-phonon spectra of the purified VK3 *cis-syn* and *cis-anti* dimers are shown in Figure 8a. The comparison with the spectra of the photoproducts obtained from the irradiation of the single crystals (Figure 5a) provides definitive evidence that irradiation of VK3-I exclusively yields the *cis-syn* isomer, while irradiation of VK3-II results unambiguously in the *cis-anti* dimer. A comparison with DFT-simulated spectra over this wavenumber range given in Figure S8 confirms that the obtained dimeric crystalline forms correspond to those reported in the literature. The assignment is straightforward for the *cis-syn* species, as the agreement with the simulation is striking. For the *cis-anti* species, although the frequency agreement is good, the comparison of band intensities may be influenced by polarization effects.

**Figure 8:**
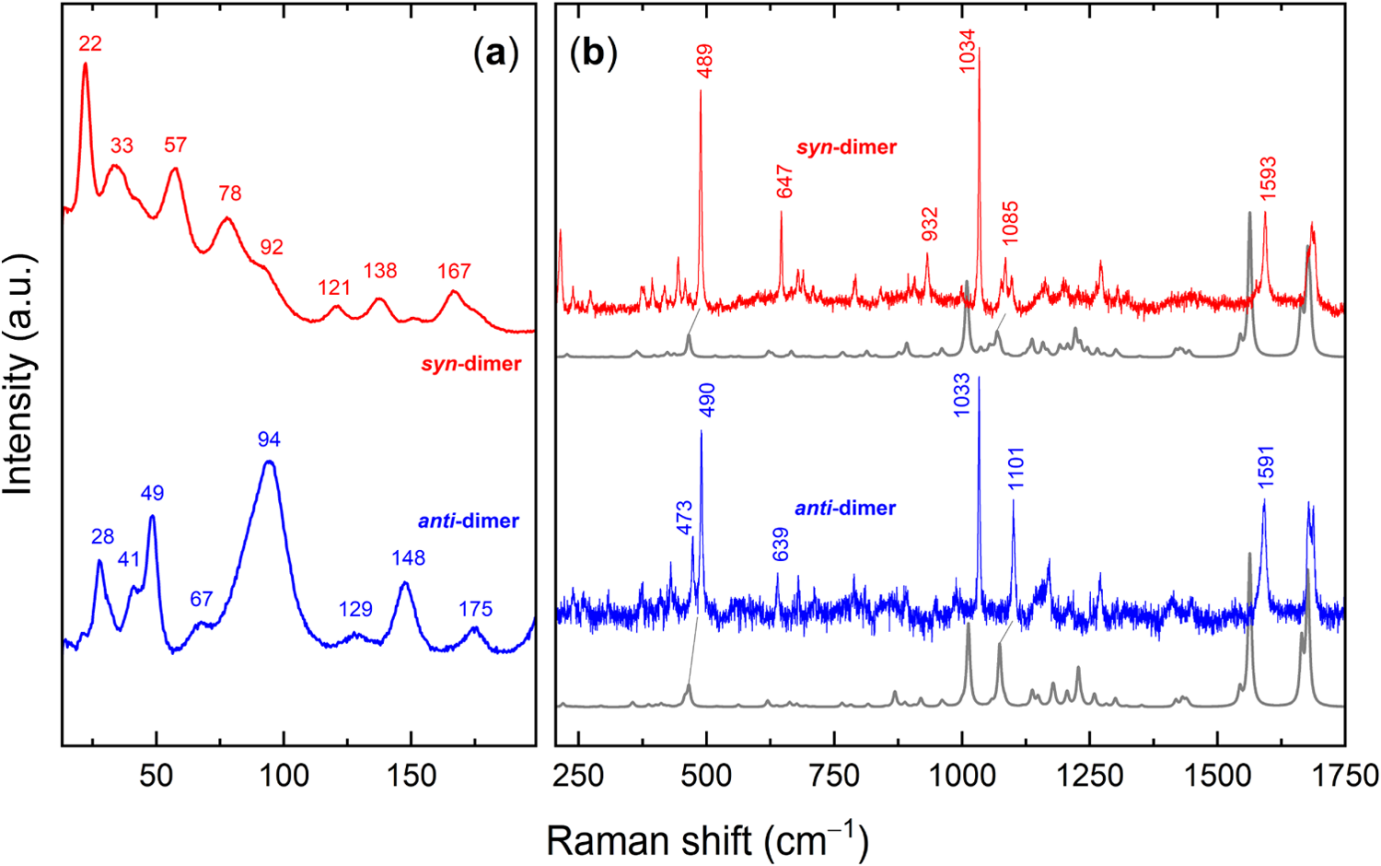
Raman spectra of the VK3 *cis-syn* and *cis-anti* dimers in the (a) lattice-phonon region and the (b) intramolecular mode region, where experiments are compared with DFT simulations.

DFT simulations of the Raman and IR spectra of the VK3 dimers as isolated molecules were performed to assist the vibrational assignment and identify spectral features of the two compounds that would allow for their discrimination. The isolated molecule comparison was preferred over solid-state intensity calculations to reduce the computational burden associated with the number of molecules in the crystal unit cells and to facilitate band assignment. The frequencies were scaled by the appropriate scaling factor (<1) to account for their systematic overestimation due to the harmonic approximation and functional choice. In this case, however, the scaling slightly increases the apparent discrepancy, as the unscaled frequencies are already very close to the experimental solid-state values. Despite this, the relative positions and intensities of the diagnostic bands identifying the dimers remain consistent. Figure 8b compares the calculated Raman spectra with the experimental results. The IR spectra, presented and discussed in Figure S9, reveal clearer differences between the two dimers. However, band broadening from unresolved crystal splitting ultimately makes their spectral features nearly indistinguishable.

### Reaction Kinetics

The kinetics of a photochemical reaction reflects its underlying mechanism.^42^ When minimal packing rearrangements are required, the reaction proceeds uniformly, and it is known to follow nearly first-order kinetics. Conversely, when significant structural rearrangements within the lattice are necessary, the process begins slowly but accelerates over time, driven by the presence and formation of defects, making cooperative or autocatalytic effects increasingly important. The simultaneous presence of lattice-phonon patterns from both the reagent and product during the irradiation of single crystals indicates that dimerization in VK3 follows a heterogeneous mechanism.^18^ A series of FTIR spectra of the powders, recorded as a function of irradiation time (Figure 6 reports a selection of them), provides insight into the kinetic law governing dimerization in this system, showing the possible role played by defects. In fact, in contrast to the selective formation of the *cis*-isomer observed in single crystals of the two polymorphs, the powder form of polymorph VK3-I leads to the formation of both stereoisomers. The spectral analysis of the powder photoproduct shows that the formed dimeric species (*cis-syn* and *cis-anti*) exhibit only slight differences in their spectroscopic properties. As a result, their spectra cannot be individually resolved at any irradiation time, where they also overlap with monomer bands. Consequently, kinetic information is dispersed across the time evolution of broad frequency regions within complex, highly congested spectra. A suitable approach to extract kinetic trends is the principal component analysis (PCA), which has been previously applied to *trans*-cinnamic acid derivatives.^19^ The method relies on eigenvalue decomposition, which represents highly correlated data as linear combinations of independent components (or momenta), ranked by their contribution to the total variance.^43,44^ In the spectra of the mixtures containing both monomers and dimers, the first PCA component typically captures many features common to both. Instead, the second component is expected to be the most informative as it distinguishes between their spectral contributions. Crucially, it encodes how the relative concentrations of monomers and dimers evolve upon irradiation, allowing for the determination of the kinetic law.^19^ Higher-order components, starting from the third, may reveal the presence of the two distinct photoisomers provided their kinetic constants and spectra differ significantly. A detailed explanation of the mathematical approach used in this work to apply PCA analysis to the spectroscopic data, and how the fractions α­(*t*) of the reactant as a function of irradiation time are directly derived from it, is provided in the Supporting Information (SI).

The PCA analysis of the FTIR spectra of Figure 6 was carried out using different kinetic models. Initially, a first-order kinetic model was assumed. This was because the mechanism derived for the photodimerization of cinnamic acid derivatives^19^ was found to satisfy such a relationship under the assumption that at any time a large population of electronically excited monomers homogeneously distributed in the crystal transforms into reacting excimers. On the other hand, the monomers in the VK3 lattice are not reciprocally oriented as favorably as in the cinnamic acids, which represent a paradigmatic homogeneous reaction. To assess whether defects play a role in a cooperative mechanism the classical Avrami equation, also known as the Johnson–Mehl–Avrami–Erofeev–Kolmogorov (JMAEK) model,^45–50^ has been applied to analyze the photodimerization kinetics of anthracene derivatives,as reported in ref 42. This equation is in fact widely used to describe solid-state transformations and predicts a sigmoidal behavior for the growth fraction α­(*t*), which represents the remaining fraction of reactant as a function of time

1 $$\alpha \left(t\right)=e^{-(k{t)}^{n}}$$

The main drawback of the JMAEK model lies in the difficulty of interpreting the physical meaning of its parameters *k* and *n*, and therefore, in this work, the Finke–Watzky (FW) model^51,52^ was adopted.

2 $$\alpha \left(t\right)=\frac{k_{1}+k_{2}}{k_{2}+k_{1}e^{{(k}_{1}+k_{2})t}}$$

The two models have been found to yield often nearly indistinguishable kinetic profiles,^52^ and the similarity arises because both can account for reaction kinetics involving an initial slow phase followed by an accelerated step due to autocatalytic effects or cooperative phenomena. However, the FW model offers parameters with clear physical interpretations, as *k*_1_ is the rate constant of the original noncatalyzed reaction and includes the initial step of nucleation, while *k*_2_ accounts for the autocatalytic/inhibitory mechanism and can, accordingly, be either positive or negative.^52^ The model has therefore been used to describe nontopotactic autocatalytic dimerization processes.^18,53^

Although a first-order fit provided a reasonable description for the VK3 data, with a rate constant of *k* = 0.0185 ± 0.0004 min^–1^, the FW law better captured the kinetics of the VK3 powder system, yielding rate constants *k*_1_ = 0.0161 ± 0.0002 min^–1^ and *k*_2_ = 0.0072 ± 0.0006 min^–1^. The difference in the Akaike Information Criterion, which compensates for the different number of parameters (AIC_FW_ – AIC_1rst_ ≈ −1), was used to compare models and determine the one to select.

The better agreement with the FW model, with a rate constant *k*_2_ smaller, but positive and still of the same order of magnitude compared to *k*_1_, suggests that cooperative effects play some role in the reaction, with structural changes within the lattice helping its acceleration. As the analyzed reaction takes place in a crystalline powder where the surface exposed to the radiation is often renewed, one may expect that defects are generated continuously throughout the process. These defects, in turn, create an increasing number of reactive sites, where molecular orientations and intermolecular distances become more favorable for the reaction to proceed. However, this very process disrupts the inherent stereoselectivity exhibited by the intact crystal lattice.

### Photochemical Pathway to the Formation of the *cis*-Isomers

As a first step in understanding the solid-state photochemical behavior of VK3, we investigated whether the selectivity observed in the formation of the *cis*-dimer isomers, as opposed to the *trans*-isomers, could be attributed to the thermodynamic factor.

The equilibrium geometries and energies of the four isomers were calculated at the B3LYP/def2-TZVP level, and the results are reported in Table S10. For the *cis*-isomers, the calculated geometries and angles are within 0.015 Å and 1° of the corresponding X-ray data,^2^ respectively. Unfortunately, to our knowledge, no literature data for the *trans*-isomers are available for comparison.

Rather surprisingly, the DFT energies of all structures lie within 3 kcal/mol, close to the estimated limit of accuracy for isomerization energies of the B3LYP functional.^54^ This finding points to a kinetic rather than a thermodynamic cause for the exclusive formation of the *cis*-isomers.

A full computational study of the photochemical [2 + 2] cycloaddition of VK3 represents a formidable task, which is outside the scope of this work. However, we have made a first step in this direction by looking for geometries and energies of the S_0_/S_1_ conical intersection or a better minimum-energy crossing point (MECI) between S_0_ and S_1_. It has been shown^35^ that TDDFT can provide accurate predictions for MECI geometries and energies, but still there are several qualitative and quantitative issues that need to be addressed before it can be routinely used for photochemical problems.^36^

The results of the TDDFT/B3LYP/def2-TZVP MECI calculations of the four isomers are reported in Table S11. Two unexpected aspects of these calculations emerged and were further investigated. Energy is the first. Like the equilibrium energies of the ground state, the MECI energies of all isomers fall in a surprisingly small range, about 2 kcal/mol. Geometry is the second. While the reactive centers of the *anti*-isomers have a regular parallelogram shape, like that of ethylene dimer^55^ with carbons C8,C9,C20,C21 nearly coplanar (see pictures in Table S10), the *sin*-isomers have a very distorted shape which can be roughly described as twisted trapezoids.

To check these questionable TDDFT results, we employed the Complete Active Space Self-Consistent Field method (CASSCF), as implemented into the Molpro program.^37^ The state-averaged first two singlet states of VK3 dimers were computed using an active space, including all electronic configurations resulting from the distribution of four electrons in four frontier orbitals (CASSCF­[4,4]). The MECI was found by minimizing the energy difference between the two states. The results of CASSCF are also reported in Table S11 and provide support to both issues raised by TDDFT. In fact, even the CASSCF MECI energies of four isomers are very similar, with a maximum difference of 3.6 kcal/mol. The *sin*-isomers have a trapezoid-like geometry but are less twisted compared to the results of the TDDFT calculations.

In summary, the exclusive formation of *cis*-dimers cannot be ascribed to significative differences in the energies of the S_0_/S_1_ conical intersections.

## Discussion and Conclusions

This study comprehensively investigated the photodimerization of VK3-I and VK3-II single crystals, showing how their distinct molecular packing influences both the photoreactivity and stereoselectivity. Using Raman spectroscopy, we tracked the transformation of the monomer to the dimer, capturing both intermolecular and intramolecular vibrational changes, which served as proxies for physical and chemical modifications. Irradiation with wavelengths at the absorption tail of the monomer at low power densities induced highly selective crystal-to-crystal transformations: VK3-I yielded the *syn*-dimer, while VK3-II formed the *anti*-dimer.

Beyond the single-crystal observations, the kinetic analysis of the VK3-I powder exhibited an autocatalytic component, unlike cinnamic acid derivatives. This suggests a defect-mediated reaction mechanism, where lattice imperfections progressively increase the concentration of reactive sites, facilitating the reaction. In powders, a significant fraction of molecules are on or near the surface of microcrystals where steric constraints are relaxed. Thus, surface molecules might adopt slightly different geometries, making easier the access to both reactive alignments (i.e., *cis-syn* and *cis-anti*).

Notably, the single-crystal transformations of VK3 do not strictly adhere to Schmidt’s topochemical postulates, indicating that photodimerization can proceed through mechanisms beyond lattice preorganization or defects. Even relatively rigid molecules can exhibit some mobility in the solid state, influenced by lattice-phonon vibrations that modulate intermolecular interactions. While defect-driven mechanisms may contribute to the photochemical behavior of the powder, the high stereoselectivity observed in single crystals suggests alternative pathways, in which specific molecular dynamics enable precise spatial control over dimerization. The importance of the crystal environment, on the other hand, is highlighted by the computational analysis of the reaction pathways, which strongly suggests that the observed selectivity in the formation of the *cis*-isomers is in fact not depending on thermodynamic factors or be ascribed to significative differences in the energies of the S_0_/S_1_ conical intersections.

These findings refine the understanding of solid-state photoreactivity and challenge the conventional dichotomy between defect-driven and purely topochemical mechanisms.

## Supplementary Material



## Notes

Crystallographic data of vitamin K3 polymorphs and dimers; DFT simulation of vibrational spectra of VK3-I and VK3-II polymorphs and dimers; experimental and calculated Vitamin K3 absorption spectra; principal components analysis of the reaction kinetics; and TDDFT and CASSCF/def2-SVP conical intersection energies (PDF)

## ⊥

CNR - National Institute of Optics (INO), Via Branze 45, 25123 Brescia, Italy

## #

CINECA, Via Magnanelli 6/3, 40033 Casalecchio di Reno, Bologna, Italy
