# Supplementary material for: Unraveling Polymorphic Control in the Solid-State [2 + 2] Cycloaddition of Vitamin K3: Insights from Single-Crystal Irradiation
Source: J Am Chem Soc. 2025 Jun 4;147(24):21109–20. doi: 10.1021/jacs.5c06303 (PMC12186518; doi:10.1021/jacs.5c06303)
Supplement: Supplementary file 1 [file ja5c06303_si_001.pdf]

Supporting Information

Unraveling polymorphic control in the solid-state [2 + 2] cycloaddiction of vitamin K_3: insights from single-crystal irradiation

Fabio Loprete[a ‡], Lorenzo Pandolfi[a,b ‡], Andrea Giunchi[a,c], Sara Pandolfi[a], Simone d'Agostino[d], Riccardo Tarroni[a], Patrizio Graziosi[e], Raffaele G. Della Valle[a], Tommaso Salzillo[a], Elisabetta Venuti*[a]

[a] Dipartimento di Chimica Industriale "Toso Montanari", Università di Bologna, Via Piero Gobetti 85, 40129 Bologna, Italy

[b] present address CNR - National Institute of Optics (INO), Via Branze 45, 25123 Brescia, Italy

[c] present address CINECA - Via Magnanelli 6/3, 40033 Casalecchio di Reno (Bologna), Italy

[d] Dipartimento di Chimica "Giacomo Ciamician", Università di Bologna, Via F. Selmi 2, 40126 Bologna, Italy

[e] CNR - Institute for Nanostructured Materials (ISMN), Via Piero Gobetti 101, 40129 Bologna, Italy

[‡] These authors contributed equally to this work.

*Corresponding Author (email: elisabetta.venuti@unibo.it)

KEYWORDS Vitamin K3, Polymorphism, [2 + 2] photodimerization, DFT, TDDFT, Vibrational Spectroscopies

Crystallographic data of VK3-I and VK3-II polymorphs and its *cis-syn* and *cis-anti* dimers

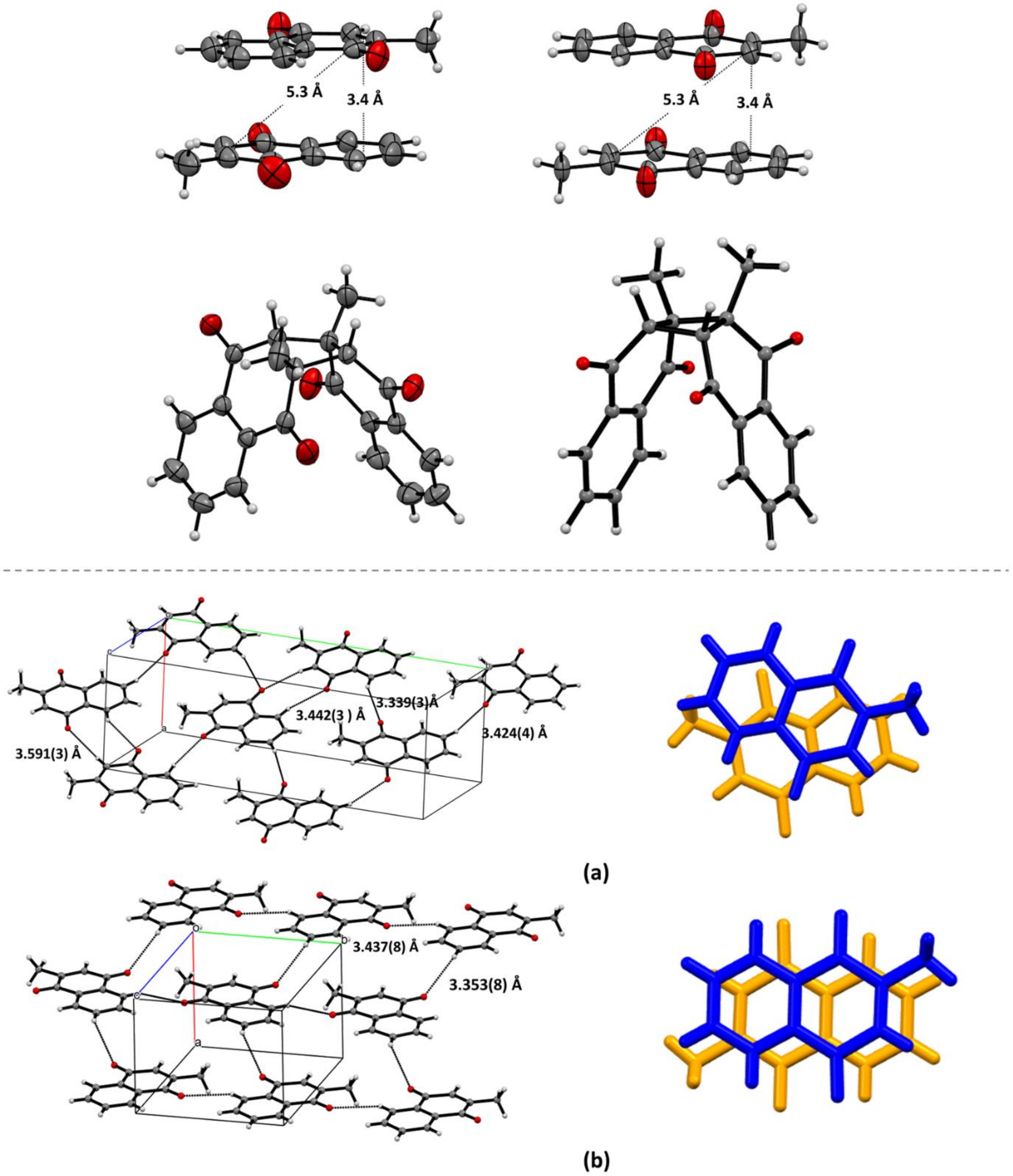

Figure S1. Top: ORTEP representations of the monomer pairs in the VK3-I (left) and VK3-II (right) polymorphs are shown in their pre-organized orientations, along with the int**er**-molecular distances between the reactive bonds within the unit cells. The resulting cis-dimers are also shown. (Note: an ORTEP representation is available only for the dimer structure solved in this work.)**; Bottom:** short C-H-O contacts and molecular layer stacking in a) VK3-I and b) VK3-II.

Table S1 Literature crystallographic data of VK_3-I and VK_3-II polymorphs.[1]

	VK3-I	VK3-II	
**Refcode**	IVEJUO02	IVEJUO03	
**Space group**	$P2_1/c$	$P2_1/c$	$P2_1/n$ Reduced Niggli Cell
**Z, Z'**	8, 2	4, 1	4, 1
**a, b, c (Å)**	7.502(5), 20.715(13), 11.168(7)	6.966(5), 8.245(6), 15.234(9)	6.966, 8.245, 14.981
**α, β, γ (°)**	90, 97.703(11), 90	90, 106.10(3), 90	90, 100.61, 90
**V (Å^3)**	1719.89	840.64	840.64
**T (K)**	297	297	
**R-factor (%)**	14.25	13.71	

Table S2 Crystallographic data of VK_3-I and VK_3-II polymorphs of this work.

	VK3-I	VK3-II
**Space group**	$P2_1/c$	$P2_1/n$
**Z, Z'**	8, 2	4, 1
**a, b, c (Å)**	7.4675(7), 20.6758(16), 11.1277(8)	6.8428(19), 8.1839(9), 14.835(3)
**α, β, γ (°)**	90, 97.877(8), 90	90, 100.76(2), 90
**V (Å^3)**	1701.87	816.165
**T (K)**	297	200
**R-factor (%)**	5.33	10.85

Table S3 Literature crystallographic data of the VK_3 dimers from reference 2 and this work.

	cis-syn dimer	*cis-anti* dimer	*cis-anti* dimer	*cis-anti* dimer[a]
**Refcode**	PIKZOY	PIKZUE	This work	This work
**Space group**	C2/c	$P\bar{1}$	C2/c	Reduced Niggli Cell
**Z, Z'**	8, 1	2, 1	4, 0,5	2, 1
**a, b, c (Å)**	10.803(2), 11.611(3), 26.827(4)	8.397(2), 8.400(2), 13.533(4)	8.7343(7), 14.3065(10), 13.5072(9)	8.3810, 8.3810, 13.5072
**α, β, γ (°)**	90, 97.40(1), 90	90.58(2), 90.70(2), 117.05(2)	90, 90.847(7), 90	90.44,90.44 117.19
**V (Å^3)**	3336.98	849.93	1687.64	849.92
**R-factor (%)**	6.0	6.9	5.0	

a) In Ref. 2, (Ref 3 of the main text) the crystallographic structure was reported in the $P\bar{1}$ space group, whereas our redetermination revealed a higher symmetry consistent with the *C*2/*c* space group. However, the previously reported $P\bar{1}$ cell closely matches the Niggli-reduced cell derived from our structure, demonstrating that the two structures are, in fact, equivalent.

[1] Rane, S.; Ahmed, K.; Salunke-Gawali, S.; Zaware, S. B.; Srinivas, D.; Gonnade, R.; Bhadbhade, M. Vitamin K3 Family Members–Part II: Single Crystal X-Ray Structures, Temperature-Induced Packing Polymorphism, Magneto-Structural Correlations and Probable Anti-Oncogenic Candidature. *J. Mol. Struct.* **2008**, *892* (1–3), 74–83.

[2] Taira, Z.; Kido, M.; Tanaka, M.; Asahi, Y. X-Ray Structures of Two Photodimers of 2-Methyl-1, 4-Naphthoquinone (Menadione). *Chem. Pharm. Bull. (Tokyo)* **1993**, *41* (12), 2183–2186. https://doi.org/10.1248/cpb.41.2183.

DFT simulation of vibrational spectra of VK3-I and VK3-II polymorphs

The vibrational spectra of both polymorphs were analyzed using the factor group approach based on unit cell symmetry. According to the **k** = 0 selection rule, only modes transforming as the totally symmetric irreducible representation of the translational symmetry subgroup are considered. These involve in-phase atomic displacements across unit cells, allowing analysis within a single unit cell classified by its factor group, isomorphic to the crystal class's point group.

For vitamin K_3 (N = 21 atoms), the degrees of freedom split into 57 vibrations, 3 rotations and 3 translations. In VK3-I (Z = 8) the total degrees of freedom are 3NZ = 504, comprising 456 intramolecular vibrations and 48 lattice-phonon modes (with 3 non-optically active acoustic modes). Raman-active modes include 114 A_g + 114 B_g intramolecular and 12 A_g +12 B_g lattice-phonon modes; infrared-active modes include 114 A_u +114 B_u intramolecular and 11 A_u +10 B_u lattice-phonon modes. Each molecular mode results in two A_g, B_g, A_u, B_u quadruplets. **Table S4** further illustrates this relationship.

VK3-II (Z = 4) follows a similar pattern, with 3NZ = 252 total degrees of freedom, divided into 228 intramolecular vibrations and 24 phonon modes (3 acoustic). Raman-active modes include 57 A_g + 57 B_g intramolecular and 6 A_g + 6 B_g lattice-phonon modes, and IR-active ones include 57 A_u + 57 B_u intramolecular and 5 A_u + 4 B_u phonon modes. Thus, each molecular mode gives rise to a single A_g, B_g, A_u, B_u quadruplet. **Table S5** further illustrates this relationship.

The clear-cut distinction between lattice phonons and intramolecular vibrations holds strictly within the rigid molecule approximation, where the coupling between normal modes associated with intermolecular interactions and those governed by the intramolecular force field is negligible. However, in a more general case, vibrational modes may exhibit varying degrees of mixing between these two contributions. The extent to which a mode retains its lattice-phonon character – *i.e.*, the fraction of rigid-body translational and rotational motion – can be quantitatively assessed in DFT vibrational analysis. This is achieved by projecting the mode eigenvectors onto the three principal molecular inertia axes, with the corresponding squared projections provided in **Table S6.**

Table S4 Symmetry correlation table of the vibrational modes of VK3-I.

**Molecular symmetry** C_s	**Site Symmetry** C_1	**Space group symmetry** $P2_1/c$
38 A' (IR, Raman) + 1R + 2T →		→ 114 A_g + 12 A_g lattice phonons (Raman)
		→ 114 B_g + 12 B_g lattice phonons (Raman)
19 A" (IR, Raman) + 2R + 1T →	→ A 57 + A 57 →	→ 114 A_u + 11 A_u lattice phonons (IR) + 1 acoustic
		→ 114 B_u +10 B_u lattice phonons (IR) + 2 acoustics

Table S5 Symmetry correlation table of the vibrational modes of VK3-II.

Molecular symmetry C_s	Site Symmetry C_1	Space group symmetry $P2_1/n$
38 A' (IR, Raman) + 1R + 2T →		→ 57 A_g + 6 A_g lattice phonons (Raman)
	→ A 57 →	→ 57 B_g + 6 B_g lattice phonons (Raman)
19 A'' (IR, Raman) + 2R + 1T →		→ 57 A_u + 5 A_u lattice phonons (IR) + 1 acoustic
		→ 57 B_u + 4 B_u lattice phonons (IR) + 2 acoustics

Table S6 Wavenumbers of the vitamin K_3 polymorphs modes calculated below 200 cm^{-1} (with symmetry). Calculated squared translational components TL, TM, TN and rotational components RL, RM, RN along and around the three inertia axes L, M, N are also given. Their sum yields the character of lattice phonon of the mode.

VK3-I polymorph								VK3-II polymorph							
Freq.	Sym.	TL	TM	TN	RL	RM	RN	Freq.	Sym.	TL	TM	TN	RL	RM	RN
-0.2	Bu	0	46	54	0	0	0	0.2	Bu	17	26	57	0	0	0
0.2	Au	98	2	0	0	0	0	0.2	Au	61	39	0	0	0	0
0.2	Bu	2	52	46	0	0	0	0.2	Bu	22	35	43	0	0	0
12.5	Ag	3	6	48	1	22	19	26.3	Bg	0	1	1	18	80	0
13.5	Au	0	0	68	1	30	0	32.5	Bu	59	37	0	1	0	2
18.1	Ag	10	11	16	1	12	49	32.5	Au	0	0	95	0	0	0
19.3	Bu	5	1	0	3	27	62	33.6	Ag	19	27	3	26	24	1
23.7	Bu	3	4	1	2	63	26	42.3	Bg	26	59	0	13	1	1
25.2	Au	6	30	1	5	0	57	43.1	Bg	46	39	3	8	3	1
26.7	Ag	87	0	1	1	1	6	43.3	Ag	48	8	6	15	20	3
29.9	Au	7	16	1	15	2	60	49.7	Bg	27	0	3	53	12	2
30.9	Bg	10	4	1	4	1	79	52.0	Ag	21	57	1	0	15	7
33.9	Bu	22	4	0	5	7	59	54.6	Au	36	56	0	1	0	6
35.0	Ag	12	5	1	3	3	73	57.6	Ag	7	5	3	24	10	49
37.6	Bg	24	16	1	3	9	47	59.3	Au	2	3	0	0	2	91
38.3	Bg	8	80	2	0	3	6	63.1	Ag	2	1	2	33	21	41
41.0	Au	39	2	1	51	4	2	66.9	Bu	1	0	0	3	1	5
43.2	Au	2	39	12	2	26	18	68.9	Bg	0	0	6	1	0	88
43.9	Ag	5	84	2	0	4	3	69.2	Au	0	0	0	63	2	0
44.9	Bu	79	4	1	1	0	14	69.7	Bu	1	1	0	69	2	19
45.4	Bg	19	1	64	1	14	1	74.7	Au	1	1	3	28	2	1
46.5	Ag	18	56	3	1	4	16	75.9	Bu	0	0	0	20	2	72
46.7	Bg	56	1	12	15	2	11	78.2	Bg	0	0	56	4	3	8
48.5	Au	13	55	4	3	10	11	78.5	Ag	3	0	54	0	5	0
50.8	Bu	26	2	0	58	2	3	100.6	Bu	0	0	0	5	88	1
51.0	Ag	53	14	2	2	5	22	100.6	Au	0	0	1	5	86	2
52.0	Bg	23	3	16	39	12	3	108.8	Bg	0	1	29	0	0	0
52.4	Au	21	28	3	15	7	25	109.4	Ag	1	2	30	0	4	0
52.7	Bu	1	60	18	7	1	11	134.9	Bu	0	0	0	1	4	0
55.3	Bg	40	5	1	34	3	15	136.6	Au	0	0	0	1	5	0
56.2	Au	11	14	14	13	25	22	137.8	Ag	0	0	2	2	0	0
56.3	Bu	35	0	3	34	2	20	139.5	Bg	0	0	2	1	1	0
57.1	Ag	3	7	26	13	40	4								
59.7	Bg	5	0	3	9	53	28								
61.3	Bg	1	70	1	4	0	6								
61.3	Ag	2	6	3	63	12	5								
65.6	Au	3	4	3	66	1	2								
69.1	Bg	6	5	2	49	7	1								
70.0	Ag	2	1	7	70	5	0								
70.6	Bu	22	0	5	62	1	0								
73.5	Ag	2	3	24	13	3	1								
74.9	Au	0	3	65	9	1	2								
75.4	Bg	6	10	6	20	2	1								
75.7	Ag	2	1	42	21	3	1								
76.6	Bg	1	2	63	10	1	0								
77.9	Bu	1	13	27	19	6	2								
78.3	Bu	1	11	20	1	7	1								

78.4	Au	0	3	5	11	5	0								
92.5	Bu	0	0	0	4	66	1								
94.3	Ag	1	2	5	2	39	1								
95.9	Au	0	2	5	3	56	1								
97.9	Bu	0	1	20	1	3	1								
100.1	Bg	1	0	1	3	68	1								
102.7	Au	0	1	14	4	17	0								
109.0	Ag	0	2	15	4	34	0								
110.2	Bg	0	1	22	4	10	0								
127.4	Au	0	0	0	1	0	0								
129.1	Bu	0	0	0	0	0	0								
129.6	Bg	0	0	0	1	0	0								
131.3	Au	0	0	0	0	0	0								
140.9	Bg	0	0	0	1	0	0								
142.3	Ag	0	0	0	2	0	0								
145.1	Bu	0	0	0	2	0	0								
147.8	Ag	0	0	0	1	0	0								
150.4	Au	0	0	2	0	4	0								
150.6	Bu	0	0	1	0	5	0								
150.9	Bg	0	0	3	0	3	0								
151.5	Ag	0	0	2	1	4	0								

Figure S2 shows the simulated Raman spectra of VK3-I and VK3-II polymorphs in the wavenumber interval 0-150 cm^{-1} used to study structural properties and photochemical transformations. The scatterings are represented as Lorentzian bands with FWHMs = 4 cm^{-1}, chosen to conform to the experimental features.

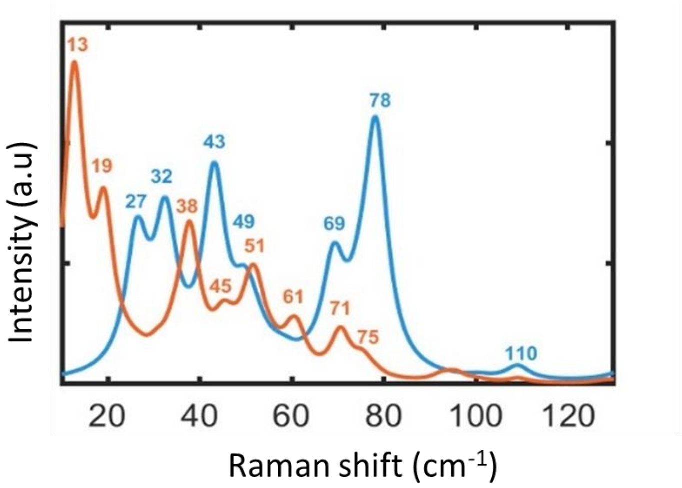

Figure S2 DFT Simulated Raman spectra of VK3-I (orange) and VK3-II (blue) polymorphs in the low wavenumber region of the lattice phonons.

Comparison with DFT-simulated IR spectra provided a good foundation for assigning the most intense vibrational bands in the crystal state. These assignments were supported by both solid-state calculations and isolated molecule simulations, which allowed for a better understanding of the vibrational pattern as a result of spectral simplification. Key assignments include the C=O stretching at 1659 cm^{-1} (simulated at 1655 cm^{-1}), the C=C double bond stretching at 1622 cm^{-1} (sim. 1610 cm^{-1}), aromatic C=C stretching at 1587 cm^{-1} (sim. 1566 cm^{-1}), aromatic C–H bending at 1261 cm^{-1} (sim. 1262 cm^{-1}) and 1234 cm^{-1} (sim. 1229 cm^{-1}), the breathing mode at 897 cm^{-1} (sim. 914 cm^{-1}), and the out-of-plane C–H aromatic bending at 777 cm^{-1} (sim. 760 cm^{-1}). The agreement between solid-state and isolated-molecule simulations underscores the reliability of these assignments, further supporting structural and kinetic analyses.

Experimental and calculated VK3 UV-Vis absorption spectra

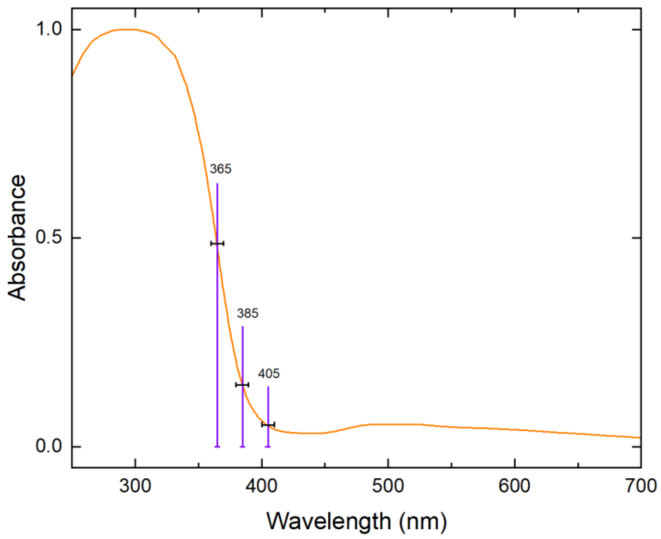

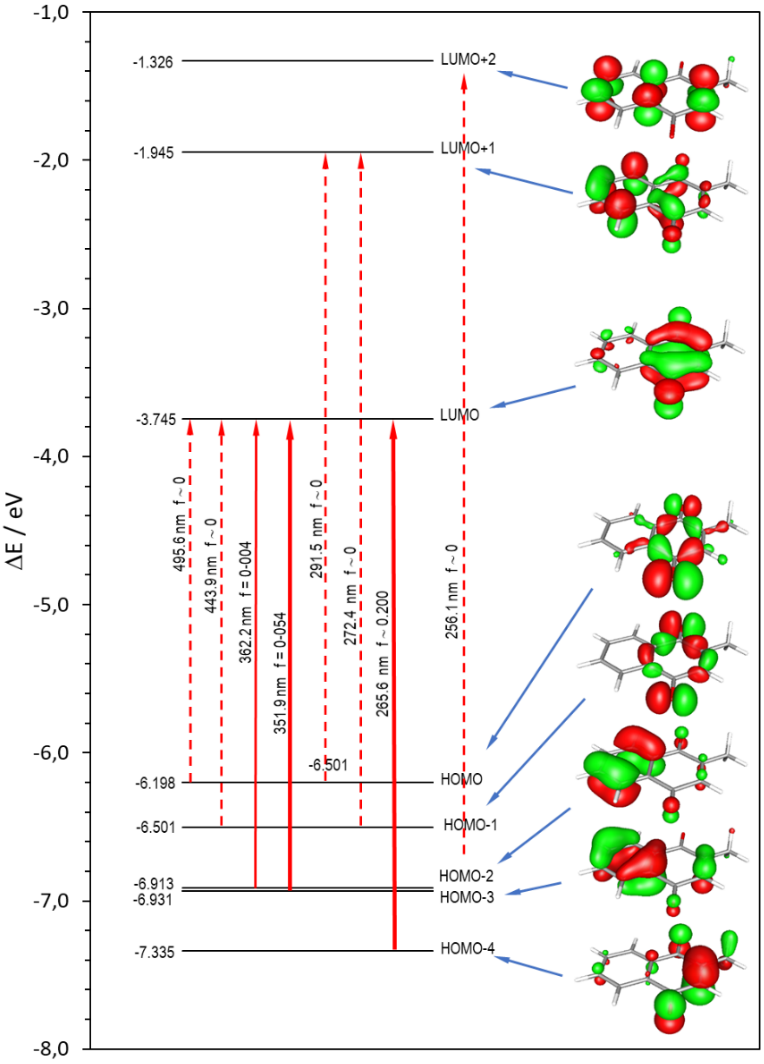

Figure S3 (top) Solid-state UV-Vis spectrum of polymorph VK3-I. The vertical lines mark the irradiation wavelengths used for the photoreaction of both polymorphs; **(bottom)** TDDFT wavenumbers and oscillator strengths of the first eight transitions, with dominant contributions shown as arrows connecting frontier orbitals.

Powder and single crystal irradiation

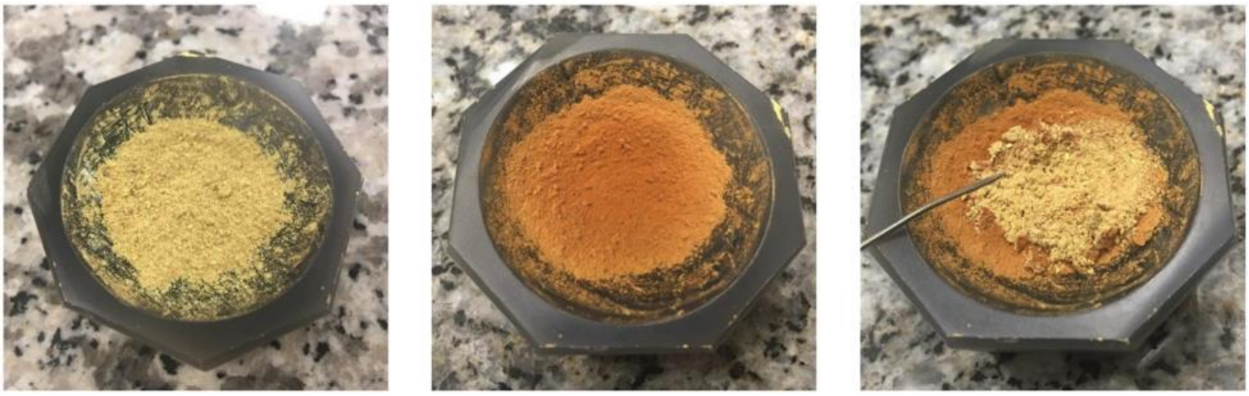

Figure S4 Images of VK3-I powder under irradiation, highlighting the surface discoloration phenomenon.

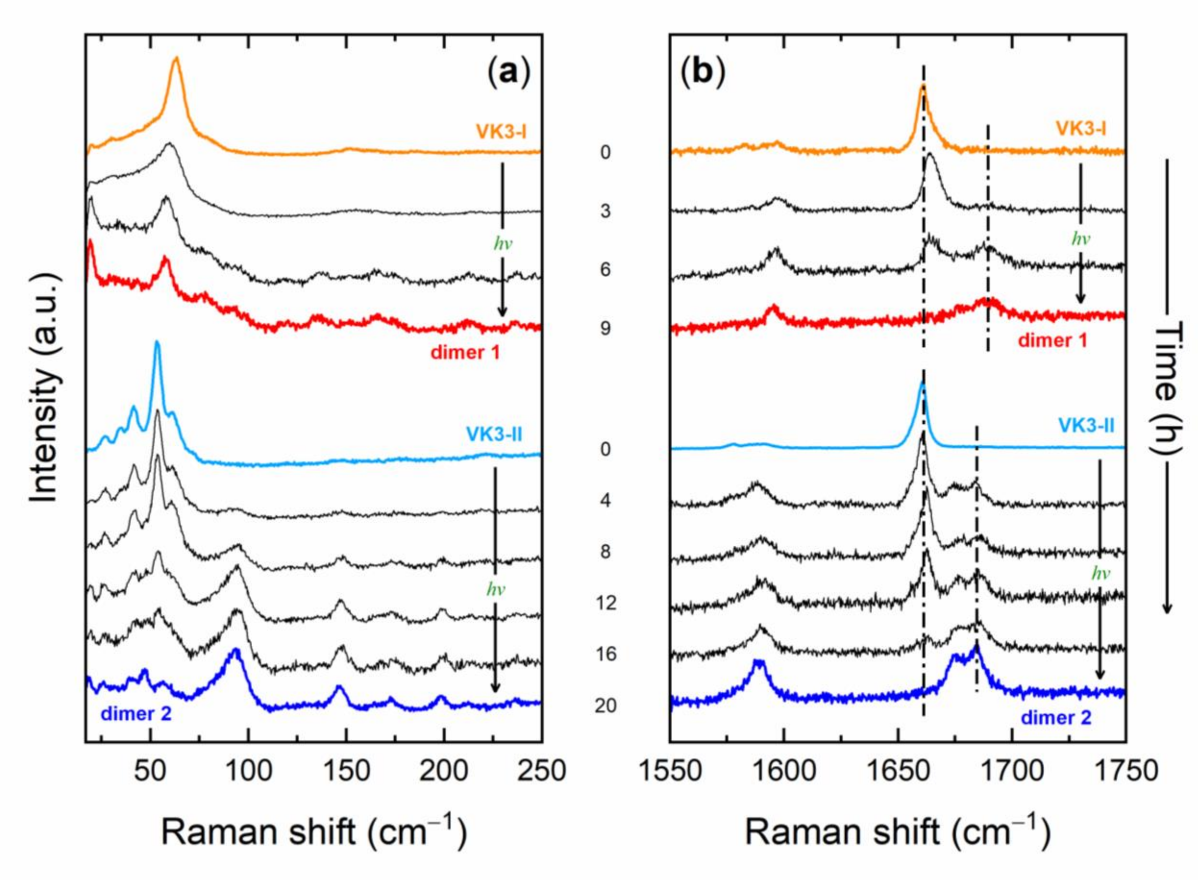

Figure S5 Raman spectra of VK3-I and VK3-II single crystals before and after irradiation with a 530 nm LED, shown for (**a**) the lattice-phonon range and (**b**) the intramolecular spectral region. The sampling intervals were 3 h and 4 h, respectively.

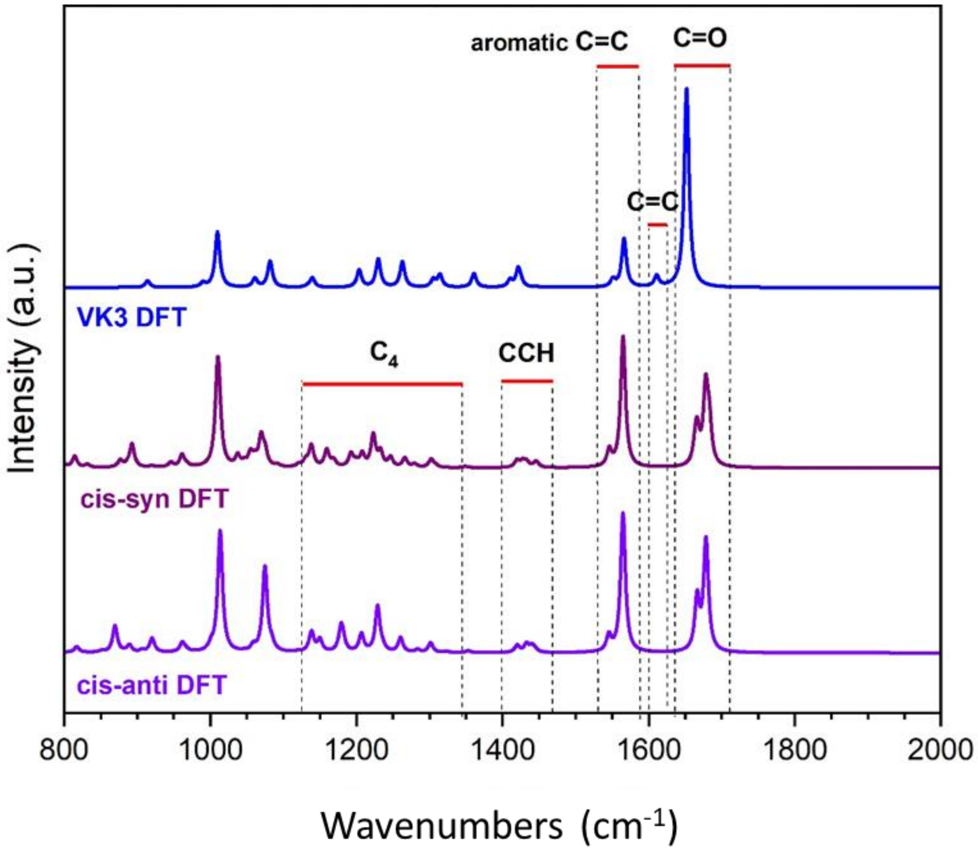

Figure S6 DFT-Simulated spectra of the VK3 monomer compared to the *cis-syn* and *cis-anti* dimers, highlighting the modes involved in the photoreaction.

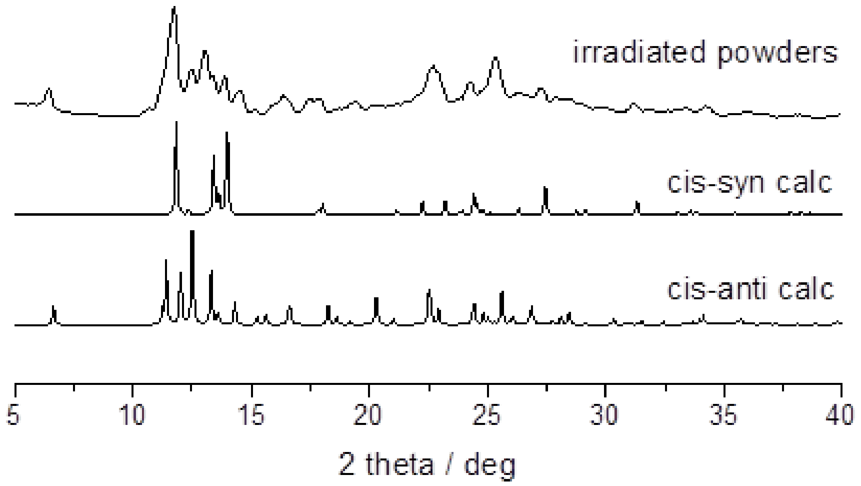

Figure S7 Comparison betweeen the experimental powder XRD pattern of the dimer mixture with simulated patterns from the literature dimer structures, confirming that the sample is a mixture of the known forms.

DFT simulation of vibrational spectra of VK3 *cis-syn* and *cis-anti* dimers

The interpretation of FTIR and Raman spectra of the photo-dimers follows the factor group approach used for monomer crystal forms.

For the *cis-syn* dimer (N = 42 atoms), vibrational degrees of freedom include 120 modes, 3 rotations, and 3 translations. In its primitive unit cell (Z = 4), the total vibrational modes amount to 3NZ = 504, comprising 480 intramolecular vibrations and 24 lattice phonons, (3 acoustic). Raman-active modes consist of 120 A_g + 120 B_g intramolecular and 6 A_g + 6 B_g lattice phonons, while IR-active modes include 120 A_u + 120 B_u intramolecular and 5 A_u + 4 B_u lattice phonons.

For the *cis-anti* dimer in its primitive cell (Z = 2), the total vibrational degrees of freedom are 3NZ = 252, with 240 intramolecular vibrations and 12 lattice phonons, including 3 acoustic branches. Raman-active modes include 120 A_g intramolecular and 6 A_g lattice phonons, while IR-active ones comprise 120 A_u intramolecular and 3 A_u lattice phonons. In this case, each molecular mode forms a doublet (A_g, A_u), whereas in the *cis-syn* dimer, it gives rise to two quadruplets (A_g, A_u, B_g, B_u). **Tables S7** and **S8** summarize these relationships.

The extent to which a mode retains lattice-phonon character can be determined via DFT vibrational analysis by projecting the mode eigenvectors onto the three principal molecular inertia axes. The corresponding squared projections are given in **Table S9**.

Table S7 Symmetry correlation table for the vibrational modes of the *cis-syn* dimer crystal structure.

**Molecular symmetry**	**Site Symmetry**	**Space group symmetry**
**C_1**	**C_1**	**C2/c**
		→ 123 A_g + 3 A_g lattice phonons (Raman)
		→ 123 B_g + 3 B_g lattice phonons (Raman)
(IR, Raman) 120 A + 3R + 3T →	→ 126 A →	
		→ 123 A_u + 2 A_u lattice phonons (IR) + 1 acoustic
		→ 123 B_u + 1 B_u lattice phonon (IR) + 2 acoustics

Table S8 Symmetry correlation table for the vibrational modes of the *cis-anti* dimer crystal structure.

**Molecular symmetry**	**Site Symmetry**	**Space group symmetry**
**C_1**	**C_1**	**$P\bar{1}$**
		→ 120 A_g + 6 A_g lattice phonons (Raman)
(IR, Raman) 120 A + 3R + 3T →	→ 126 A →	
		→ 120 A_u + 3 A_u lattice phonons (IR) + 3 acoustics

Table S9 Wavenumbers of the *cis-syn* and *cis-anti* dimer modes calculated below 200 cm^{-1} (with symmetry). Calculated squared translational components TL, TM, TN and rotational components RL, RM, RN along and around the three inertia axes L, M, N are also given. Their sum yields the character of lattice phonon of the mode.

cis-syn dimer								*cis-anti* dimer							
**Freq.**	**Symm.**	**TL**	**TM**	**TN**	**RL**	**RM**	**RN**	**Freq.**	**Symm.**	**TL**	**TM**	**TN**	**RL**	**RM**	**RN**
-0.2	Au	0	19	81	0	0	0	0.1	Bu	99	0	1	0	0	0
0.1	Bu	100	0	0	0	0	0	0.1	Bu	1	0	99	0	0	0
0.2	Bu	0	81	18	0	0	0	0.2	Au	0	100	0	0	0	0
15.7	Au	82	1	0	0	0	5	21.6	Bu	0	0	0	68	0	23
19.9	Bu	0	16	69	10	1	0	25.7	Ag	0	94	0	0	4	0
21.3	Bg	55	6	0	1	2	10	29.4	Bg	14	0	9	45	0	27
22.1	Ag	0	11	56	26	1	0	37.4	Au	0	0	0	0	81	0
32.6	Au	0	2	1	76	0	2	37.9	Bg	6	0	50	24	0	15
32.8	Bu	0	1	2	8	12	25	47.2	Ag	0	1	0	0	26	0
34.1	Bg	7	8	41	2	15	3	49.4	Au	0	0	0	0	6	0
34.2	Ag	31	0	5	14	10	1	51.9	Bu	0	0	0	16	0	73
37.2	Au	0	46	10	0	17	4	64.0	Bg	10	0	3	4	0	19
37.7	Bg	9	39	0	5	38	0	66.3	Bu	0	0	0	16	0	2
41.2	Bu	0	0	1	7	40	0	66.4	Bg	61	0	10	8	0	18
42.1	Bg	16	1	10	17	4	0	77.6	Au	0	0	0	0	10	0
42.6	Ag	28	15	0	0	50	0	84.7	Ag	0	3	0	0	53	0
44.6	Bu	0	1	4	68	22	2	96.0	Ag	0	1	0	0	9	0
45.2	Bg	2	36	4	25	3	0	96.6	Bg	6	0	26	17	0	19
46.0	Ag	19	4	6	9	0	4	106.7	Bu	0	0	0	0	0	0
47.5	Au	1	21	5	1	62	1	114.5	Au	0	0	0	0	1	0
49.1	Bu	0	0	0	1	11	68	122.5	Bg	0	0	2	1	0	0
52.1	Bg	9	1	4	1	13	65	123.1	Ag	0	0	0	0	1	0
52.5	Ag	16	5	0	11	16	7	129.4	Bu	0	0	0	0	0	0
54.4	Au	3	6	1	1	9	22	135.8	Au	0	0	0	0	1	0
56.5	Au	4	4	1	19	0	35	143.4	Ag	0	0	0	0	5	0
56.9	Ag	1	30	2	5	6	48	144.3	Bg	0	0	0	0	0	2
59.1	Bg	0	5	33	14	19	18	153.5	Au	0	0	0	0	1	0
60.4	Bu	0	1	5	3	7	2								
62.2	Bg	1	0	0	16	0	2								
65.5	Au	6	1	0	2	8	27								
68.8	Ag	1	29	12	1	13	35								
72.1	Bu	0	0	1	1	5	0								
75.1	Ag	1	1	16	31	1	1								
79.9	Bg	0	2	5	14	3	0								
89.6	Au	4	0	0	0	0	2								
89.9	Ag	3	0	0	0	0	2								
105.0	Bu	0	0	0	1	1	2								
107.4	Bg	0	1	0	0	2	2								
110.3	Au	0	0	0	0	1	1								
114.2	Ag	0	3	0	0	2	1								
116.9	Bg	0	0	0	0	1	0								
117.0	Bu	0	0	0	0	0	0								
124.0	Bu	0	0	0	0	0	0								
125.6	Au	0	0	0	0	0	0								
130.0	Bg	0	0	0	1	0	0								
132.7	Ag	0	0	2	2	0	0								
134.2	Bu	0	0	0	0	0	1								
136.4	Au	0	0	0	0	0	0								
137.7	Bg	0	0	1	2	0	0								
138.6	Ag	0	1	0	0	0	0								
139.6	Ag	0	0	0	1	0	0								
139.7	Au	0	0	0	0	1	0								
151.8	Bu	0	0	0	0	0	0								

Figure S8 shows the simulated Raman spectra of *cis-syn* and *cys-anti* dimers in the wavenumber interval 0-150 cm^{-1} used to study structural properties and photochemical transformations. The scatterings are represented as Lorentzian bands with FWHMs = 4 cm^{-1}, chosen to conform to the experimental features.

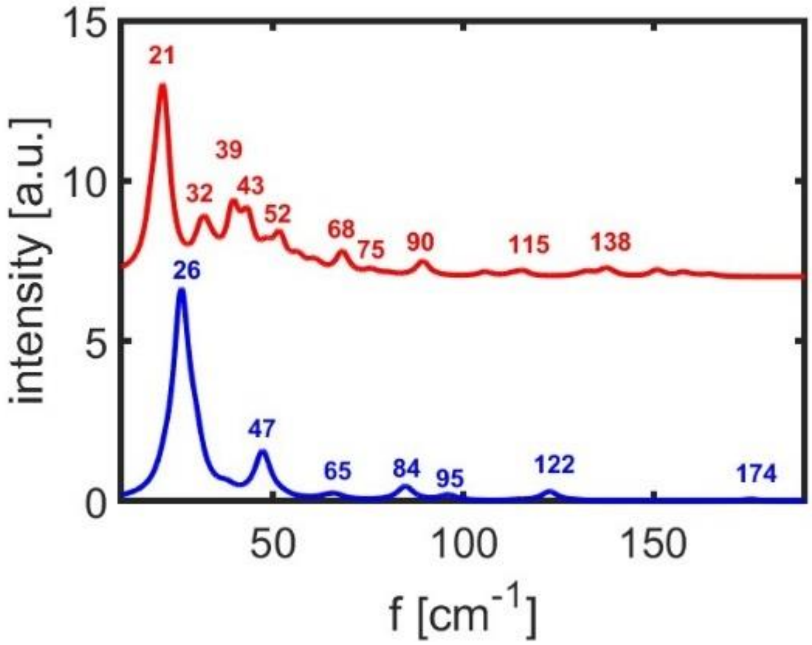

Figure S8 DFT Simulated Raman spectra of the *cis-syn* (red) and *cis-anti* (blue) dimers in the low wavenumber region of the lattice phonons.

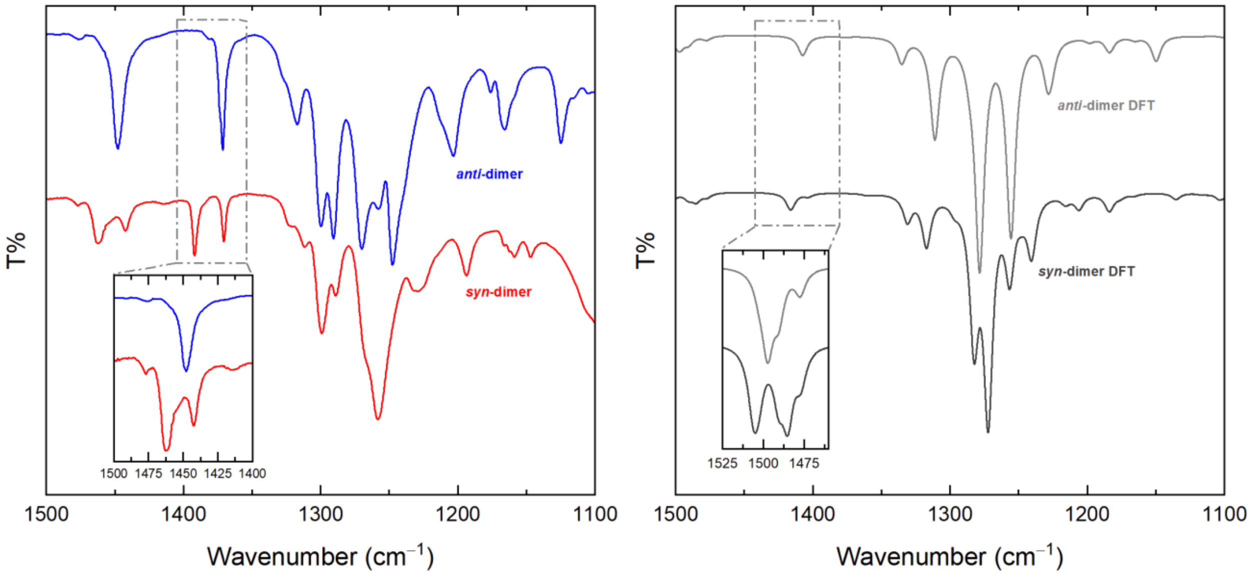

Figure S9 Experimental (left) and simulated (right) infrared spectra of the *cis-syn* and *cis-anti* dimers.

Figure S9 presents the 1100–1500 cm^{-1} fingerprint region of the experimental and calculated IR spectra of the VK3 dimers. The insets highlight the CH_3 bending modes of the four-membered rings around 1450 cm^{-1}, which differ between the *cis-syn* and *cis-anti* dimers in both experiments and simulations. Once band broadening due to unresolved crystal splitting is considered, the spectral features of the two species become nearly indistinguishable.

Fitting kinetic equations to results of Principal Components Analysis

As discussed in the main text, we have followed the photoreaction of the VK3-I crystal polymorph in powder form by repeatedly recording the FTIR spectra of a sample under continuous irradiation. We have thus obtained a collection of 22 spectra $Y_i(\omega)$ with $i = 0,1, \dots n$, which represent the intensity Y as a function of the frequency ω at increasing times $t_i = t_0, t_1, \dots t_n$. We have extracted quantitative information on the progress of the reaction through a Principal Component Analysis (PCA) of the spectra. The method represents the highly correlated spectra as a function of time as linear combinations of independent components, ranked by their importance. Following common PCA procedures, each spectrum is first standardised by subtracting its mean (effectively removing any flat background) and then normalised by dividing by its standard deviation (a step which is not mathematically compulsory for the PCA but which in our case, as we have verified by analysing synthetic datasets, helps to compensate fluctuations in overall intensity). By diagonalising either the correlation matrix or the covariance matrix (depending on the presence, or not, of the normalisation step) we thus decompose the standardised spectra $Y_i(\omega)$ in terms of a basis of orthonormal spectra $V_j(\omega)$:

$$Y_i(\omega) = V_0(\omega)\ c_{0i} + \cdots + V_n(\omega)\ c_{ni} \tag{1}$$

The coefficients $c_{0i}, c_{1i}, \dots, c_{ni}$ are in order of decreasing importance and the first few of them are plotted in **Figure S10**. The first two components alone account for almost all the overall variance of the spectra:

$$Y_i(\omega) \approx V_0(\omega)\ c_{0i} + V_1(\omega)\ c_{1i} \tag{2}$$

Next, the evolution of the chemical reaction over time is modeled under the assumption that the spectra $Y_i(\omega)$ can be described by a weighted sum of the spectra of the reagents A, $S_A(\omega)$ (the spectrum at time $t = 0$) and of the products B, $S_B(\omega)$ (the spectrum at time $t = \infty$): $Y_i(\omega) \approx S_A(\omega)\ \alpha_i + S_B(\omega)\ (1 - \alpha_i) \propto S_A(\omega)\ a_i + S_B(\omega)\ b_i$

The two components c_{0i} and c_{1i} encode the spectral features common to both reagent and products, and their differences. The remaining components account for less than $\sigma^2_{\mathrm{PCA}} = 0.55\%$ of the variance and represent the noise in the spectra. This exemplifies PCA's dimensionality reduction, condensing spectral data from thousands of frequency channels into two variables. The spectra $Y_i(\omega)$ as a function of time t_i are thus represented by the two coefficients c_{0i} and c_{1i}, that correspond to the 2D *column* vector

$$\mathbf{C}_i = \begin{pmatrix} c_{0i} \\ c_{1i} \end{pmatrix} \tag{3}$$

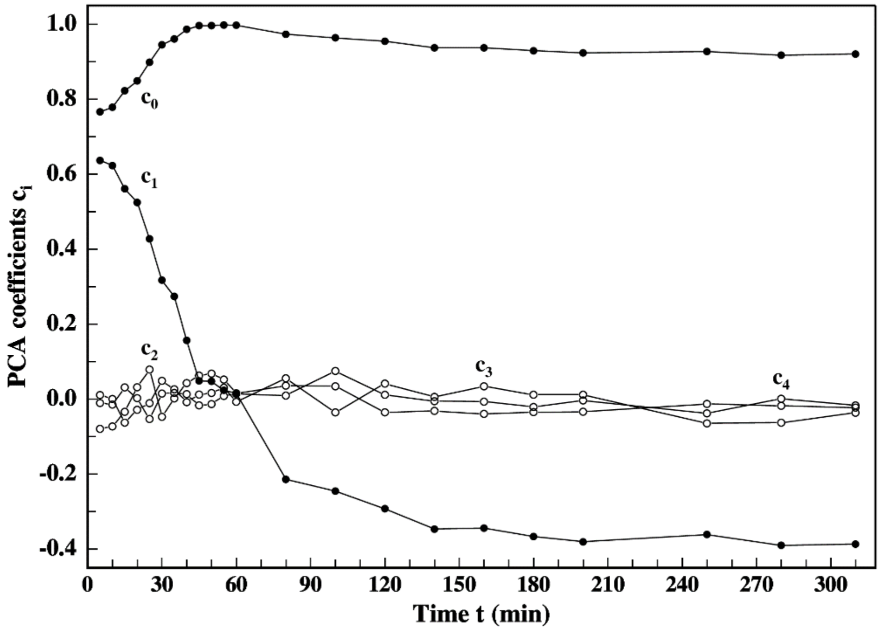

Figure S10 PCA coefficients $c_{0i}, c_{1i}, \ldots, c_{4i}$ as a function of time t_i. Since all input spectra are normalised to unit variance, the coefficients are normalised as $c_{0i}^2 + c_{1i}^2 + \cdots + c_{ni}^2 = 1$. Only coefficients c_{0i} and c_{1i} are significant (filled circles), while all remaining coefficients are small and fluctuate around zero (empty circles)

The trajectory of the 2D point $\mathbf{C}_i$ in the c_0, c_1 plane is illustrated in **Figure S11**. Since all spectra are normalized to unit standard deviation (and thus unit variance), all $\mathbf{C}_i$ points lie very close to the unit circle with radius $r = c_{0i}^2 + c_{1i}^2 = 1$. The small deviations from the unit circle are due to the noise σ_{PCA}^2.

Next, the evolution of the chemical reaction over time is modelled under the assumption that the spectra $Y_i(\omega)$ can be described by a weighted sum of the spectra of the reagents A, $S_A(\omega)$ (the spectrum at time $t = 0$) and of the products B, $S_B(\omega)$ (the spectrum at time $t = \infty$):

$$Y_i(\omega) \approx S_A(\omega)\ \alpha_i + S_B(\omega)\ (1 - \alpha_i) \propto S_A(\omega)\ a_i + S_B(\omega)\ b_i \tag{4}$$

where a_i and b_i are time-dependent coefficients. These coefficients are assumed to be proportional to the fraction α_i of reagent A remaining at time t_i and to its complement $1 - \alpha_i$. A mixture of several reagent species may be present, but we assume that their relative proportions do not change, and the same for the products. Otherwise, two spectra $S_A(\omega)$ and $S_B(\omega)$ would not be sufficient to follow the progress of the reaction. By estimating a_i and b_i, the fraction of reagents remaining at time t_i is determined as:

$$\alpha_i = \frac{a_i}{a_i + b_i} \tag{5}$$

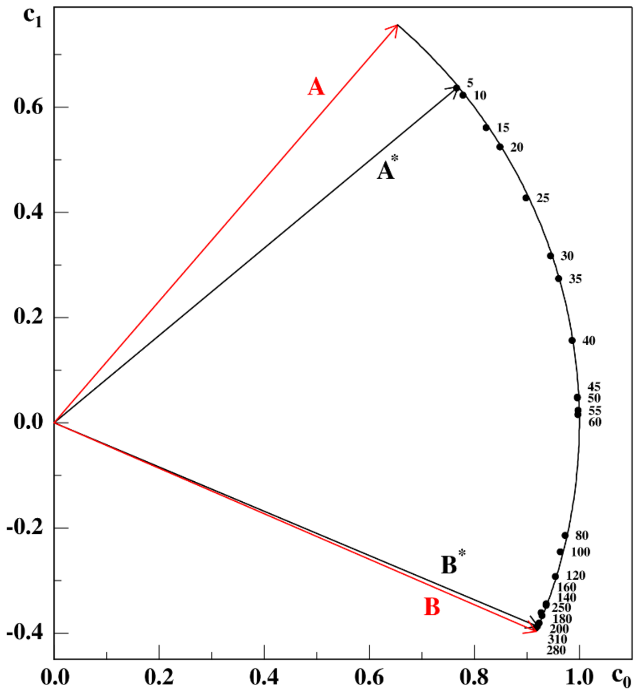

Figure S11 Dots: points $C_i = (c_{0i}, c_{1i})$ representing the spectra at the times $t_i = t_0, t_1, \ldots t_n$ (in minutes) indicated by the labels. To avoid overlaps in the drawing some labels are vertically shifted, while maintaining their non monotonic order. Arrows: normalised vectors representing the spectra of reagents and products, fixed to initial and final points ($A^* \propto C_0$ and $B^* \propto C_n$) or determined by the fit (A and B). Curve: circular arc with unit radius $r = c_{0i}^2 + c_{1i}^2 = 1$. The barely visible deviations of the points C_i from this arc are due to the noise σ_{PCA}^2. All points C_i lie in the positive quadrant spanned by the vectors A and B but, also due to the noise, not in the quadrant spanned by A^* and B^*.

Fluctuations in the overall intensity of the spectra and other possible time-dependent factors independent of the species all cancel out by computing the ratio in α_i. The fraction $\alpha(t)$ starts at 1, and decreases during the reaction, with a kinetic time law to determine. By fitting the kinetic expression $\alpha(t)$ to the estimated values of α_i, the kinetic law is verified, and the optimal parameters, such as the relative rate constant k, are determined.

To find the weights a_i and b_i we need the reference spectra $S_A(\omega)$ and $S_B(\omega)$ or, equivalently, the two normalised 2D column vectors which represent them:

$$\mathbf{A} = \begin{pmatrix} c_{0A} \\ c_{1A} \end{pmatrix} \quad \text{and} \quad \mathbf{B} = \begin{pmatrix} c_{0A} \\ c_{1A} \end{pmatrix} \tag{6}$$

Both reference vectors have unit length since the corresponding spectra are standardised and normalised (zero mean and unit variance). If reference spectra are not measured directly, reagents and products can be approximated by the initial and final standardized spectra $Y_0(\omega)$ and $Y_n(\omega)$, represented by the normalised vectors $\mathbf{A}^* \propto \mathbf{C}_0$ and $\mathbf{B}^* \propto \mathbf{C}_n$. As we will see, other choices are preferable. In any case, the known spectrum at time t_i is represented by the vector

$$\mathbf{C}_i = \begin{pmatrix} c_{0i} \\ c_{1i} \end{pmatrix} = \mathbf{A}\, a_i + \mathbf{B}\, b_i = \begin{pmatrix} c_{0A} \\ c_{1A} \end{pmatrix} a_i + \begin{pmatrix} c_{0B} \\ c_{1B} \end{pmatrix} b_i = \begin{pmatrix} c_{0A} & c_{0B} \\ c_{1A} & c_{1B} \end{pmatrix} \begin{pmatrix} a_i \\ b_i \end{pmatrix} \tag{7}$$

By inverting the matrix

$$\mathcal{M} = \begin{pmatrix} c_{0A} & c_{0B} \\ c_{1A} & c_{1B} \end{pmatrix} \tag{8}$$

we may readily obtain the weights:

$$\begin{pmatrix} a_i \\ b_i \end{pmatrix} = \mathcal{M}^{-1} \begin{pmatrix} c_{0i} \\ c_{1i} \end{pmatrix} \tag{9}$$

and, from these, the fraction of reagent remaining at time t_i, $\alpha_i = a_i/(a_i + b_i)$. The validity of the expression for α_i depends on all weights a_i and b_i being positive. This condition is satisfied if all vectors $\mathbf{C}_i$ have positive components if decomposed on the basis vectors **A** and **B**, which means that all vectors $\mathbf{C}_i$ lie in the positive non-orthogonal quadrant spanned by **A** and **B** (see **Figure S11**). Of course, this is a condition that any acceptable pair of reference vectors should satisfy. Due to the experimental noise in the spectra recorded in the present work, as shown in **Figure S11** and as discussed below, this condition is only approximately satisfied if using the vectors $\mathbf{A}^*$ and $\mathbf{B}^*$ representing the initial and final standardised spectra $Y_0(\omega)$ and $Y_n(\omega)$. Beside such possible problems with the noise, using only the initial and final $\mathbf{C}_i$ vectors to build the two reference vectors is not optimal since the initial and final spectra need not to be really at times $t = 0$ and $t = \infty$ and since all other $\mathbf{C}_i$ vectors also contain information on the spectra of reagents and products. Since the reagent fractions α_i will ultimately be fitted to a time law $\alpha(t)$ containing adjustable parameters, it makes sense to use the elements of the matrix $\mathcal{M}$ or even better of its inverse $\mathcal{M}^{-1}$, as further adjustable parameters. We cannot really adjust independently the four elements of $\mathcal{M}^{-1}$ since to two vectors **A** and **B** (which constitute the two columns of $\mathcal{M}$) have unit norm. The two rows of $\mathcal{M}^{-1}$, therefore, are also 2D vectors with unit norm. We parameterise each such (x, y) unit vector in the simplest way, as a $(\sin\tau, \cos\tau)$ pair with the angle $\tau = \arctan x/y$ as the fit parameter.

The fitting procedure for the Finke-Watzky kinetic law

To extract kinetic information from time-dependent spectra, we start with PCA decomposition of the standardized spectra (zero mean, unit variance), yielding the coefficients c_{0i} and c_{1i} for all times t_i. The coefficients for the initial and final time $i = 0$ and $i = n$ form the *initial* matrix $\mathcal{M}$ (eq. 8), from which we obtain the initial inverse matrix $\mathcal{M}^{-1}$ and, from its two rows, the initial values of its two angle parameters τ. In the opposite direction, for any given pair of τ angles we may readily obtain $\mathcal{M}^{-1}$, then all weights a_i and b_i (eq. 9), and all fractions α_i (eq. 5). To verify a possible kinetic law $\alpha(t)$ we therefore numerically minimise the sum of the squares of the "residuals" $\alpha(t_i) - \alpha_i$, by varying all adjustable parameters in the model for $\alpha(t)$ and the two τ angles which control the rows of $\mathcal{M}^{-1}$. As discussed in the main text, we have considered several kinetic laws, and ultimately settled for the Finke-Watzky (FW) model

$$\alpha(t) = \frac{k_1 + k_2}{k_2 + k_1 \mathrm{e}^{(k_1+k_2)t}} \tag{10}$$

The fraction $\alpha(t)$ of remaining reagent as a function of time t resulting from the fit is shown in **Figure S12**. The root mean square (RMS) deviation between model $\alpha(t_i)$ and fitted α_i (the residuals), which effectively describe the uncertainties on the fractions α, is $\sigma_\alpha = 0.025$.

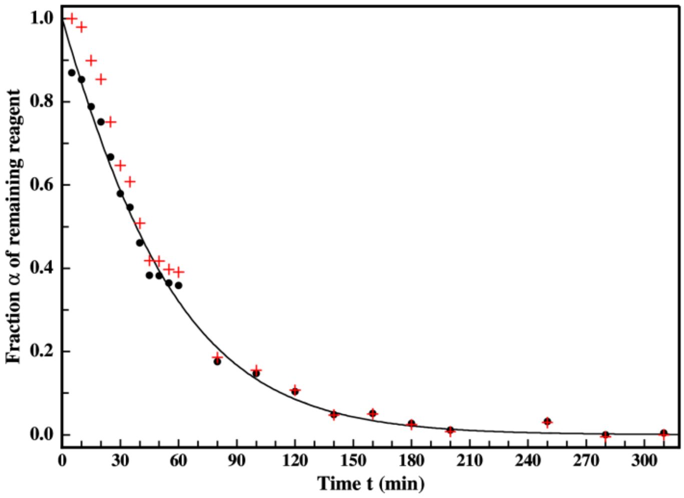

Figure S12 Fraction α of reagent at time t. Crosses and circles indicate α_i from the PCA with the two reference vectors (representing reagent and product) either fixed by the initial and final spectra (**A*** and **B***) or adjusted in the optimisation (**A** and **B**), respectively. This optimisation, with the FW model, also yields the rate constants $k_1 = 0.0161\ \mathrm{min}^{-1}$ and $k_2 = 0.0072\ \mathrm{min}^{-1}$ of the fitted $\alpha(t)$ (continuous curve).

The fitted points α_i depend on the fit itself, as they are influenced by the adjustable τ angles that define reference vectors $\boldsymbol{A}$ and $\boldsymbol{B}$, which in turn determine the reference spectra. However, this dependence is not significant. The fractions α_i^*, (**Figure S12**, crosses) obtained using fixed references vectors **A*** and **B*** as initial and final spectra $Y_0(\omega)$ and $Y_n(\omega)$, closely match the adjusted α_i (circles of **Figure S12**), confirming their reliability, with differences in expected directions. By construction, α_i^* is 1 at t_0 and 0 at t_n, though in some reagent might be already consumed at t_0 and some be left at t_n. Due to experimental noise and the constraint $\alpha_n^* = 0$, unphysical values ($\alpha_i^* < 0$) may appear, as it happens at $t_i = 280$ min. This is reflected **in Figure S11**, where the corresponding $\boldsymbol{C}_i$ point lies outside the quadrant defined by vectors A^* and B^*. When A and B are adjusted in the fit, these issues disappear: all weights a_i and b_i remain positive, and all fractions fall within the correct range ($0 < \alpha_i < 1$), further validating the procedure.

Since the full model describing the spectra as a function of time is highly non-linear, the classical coefficient of determination R^2 is inadequate for model comparison. Instead, as mentioned in the main text, we use the Akaike information criterion (AIC),[3] which combines fit quality (total squared error) with a principle of parsimony, penalizing excess of adjustable parameters. While first-order kinetics $\alpha(t) = e^{-kt}$ provides a reasonable fit for the present data, the Finke-Watzky model (eq. 10) performs significantly better, justifying the extra parameter by yielding a lower AIC.

Standard linear uncertainty estimates also fail due to nonlinearity, so we employed a parametric bootstrap resampling,[4] in which the fitted model and estimated statistical distributions are used to generate multiple synthetic datasets, thus analyzed as the experiment. In the procedure, the optimal parameters and reference vectors ($k_1, k_2, \boldsymbol{A}, \boldsymbol{B}$) are first determined. Using the reference vectors and the PCA basis vectors $V_0(\omega)$ and $V_1(\omega)$, we reconstruct the initial and final reference spectra: $S_{A,B}(\omega) = V_0(\omega)\ c_{0A,B} + V_1(\omega)\ c_{1A,B}$ (eq. 6). Synthetic spectra $Y_i(\omega) = S_A(\omega)\alpha_i + S_B(\omega)(1-\alpha_i)$ are generated at the experimental time points using the

[3] Burnham, K. P.; Anderson, D. R. Multimodel Inference: Understanding AIC and BIC in Model Selection. *Sociol. Methods Res.* **2004**, *33* (2), 261–304. https://doi.org/10.1177/0049124104268644.
[4] Dekking, F. M.; Kraaikamp, C.; Lopuhaä, H. P.; Meester, L. E. *A Modern Introduction to Probability and Statistics: Understanding Why and How*; Springer Texts in Statistics; Springer London, 2006.

FW computed $\alpha_i = \alpha(t_i)$. Applying our fitting procedure *exactly* reproduces the input rate constants, validating the approach. Finally, we introduce spectral noise to assess its impact. To estimate uncertainties in the rate constants, we generated hundreds of synthetic datasets by adding two sources of normal (Gaussian) noise: one reproducing the spectral noise σ^2_{PCA} and another perturbing $\alpha(t)$ based on the RMS fit residuals σ_α (i.e. the uncertainties on the fractions α). The fitting procedure was then repeated for all datasets. The resulting rate constants k_1 and k_2 are shown in the scatter plot of **Figure S13**, representing the expected distribution from repeated measurements and analyses (parametric bootstrap resampling). As the results of this procedure depends on model accuracy, we assessed its reliability using the jackknife (leave-one-out) method[5] as a non-parametric form of resampling. One spectrum $Y_i(\omega)$ at the time was systematically excluded from the experimental dataset, and the rate constants were recalculated based on the remaining data. The resulting estimates are also shown in **Figure S13**.

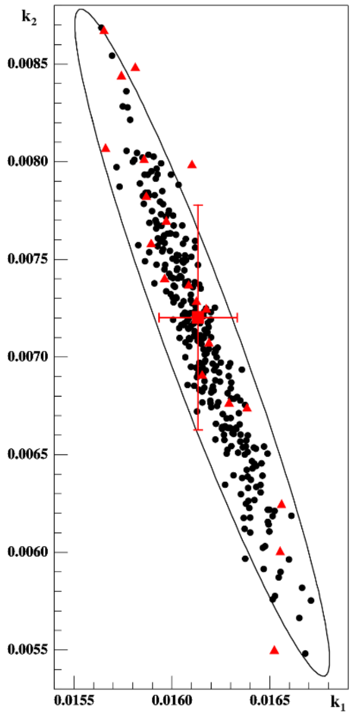

Figure S13 Optimal rate constants k_1 and k_2 (min^{-1}) fitted to various datasets. Filled circles: constants fitted to the synthetic datasets (parametric bootstrap resampling). Triangles: constants fitted to the "leave one out" datasets (non-parametric jackknife resampling). Square: constants fitted to the experimental dataset, with error bars (1σ) from the bootstrap. Elipse: 3σ confidence region from the bootstrap.

The rate constant distribution from jackknife resampling, though slightly more dispersed, largely overlap with that from bootstrap resampling, confirming the reliability of the latter in estimating uncertainties and confidence region. The standard deviation from bootstrap resampling provides the standard errors on the optimal constants: $k_1 = 0.0161 \pm 0.0002 \text{ min}^{-1}$, $k_2 = 0.0072 \pm 0.0006 \text{ min}^{-1}$. **Figure S13** clearly shows that k_1 and k_2 are strongly anti-correlated, with correlation coefficient $r_{k_1 k_2} = -0.9492$ and linear slope $\beta = \frac{dk_2}{dk_1} = -2.7239$. As expected from the functional form of the rate law (eq. 10), a change in k_2 can

[5] Babamoradi, H.; van den Berg, F.; Rinnan, Å. Bootstrap Based Confidence Limits in Principal Component Analysis — A Case Study. *Chemom. Intell. Lab. Syst.* **2013**, *120*, 97–105. https://doi.org/10.1016/j.chemolab.2012.10.007.

be partially compensated by an opposite smaller change in k_1. This correlation can be removed via singular value decomposition, which corresponds to rotate (k_1, k_2) onto new axes along $(1, \beta)$ and $(-\beta, 1)$, that is the slope and its orthogonal. Projecting the data onto these axes, once they have been normalized, yields two uncorrelated variables, whose standard deviations σ define the 3σ elliptic confidence region in Figure S13. For a normal distribution, this region contains 99.7% of the values, and, by Chebyshev's inequality,[4] it includes nearly all values even for any non-normal distribution with finite-variance. Throughout this region, k_2 remains positive, consistent with the autocatalytic nature of the FW kinetic law.

[4] Dekking, F. M.; Kraaikamp, C.; Lopuhaä, H. P.; Meester, L. E. *A Modern Introduction to Probability and Statistics: Understanding Why and How*; Springer Texts in Statistics; Springer London, 2006.

Table S10 Equilibrium energies and geometrical parameters for the bridging region of dimers, calculated at B3LYP/def2-TZVP level. The experimental values from X-ray measurements (Taira *et al.* 1993) are reported in parentheses. ΔE are differences between the equilibrium energy of each isomer and the corresponding energy of the trans-anti isomer, taken as reference.

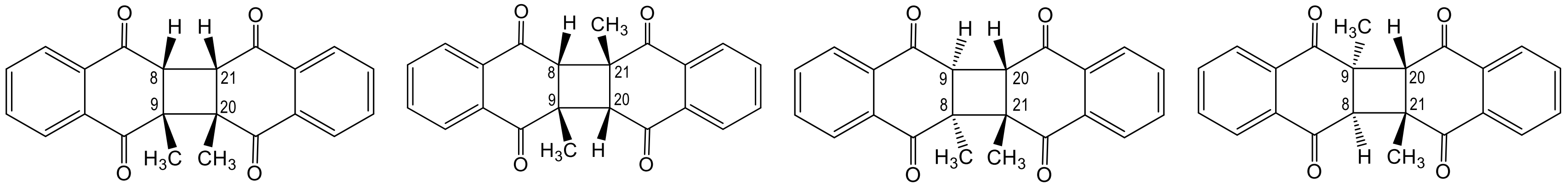

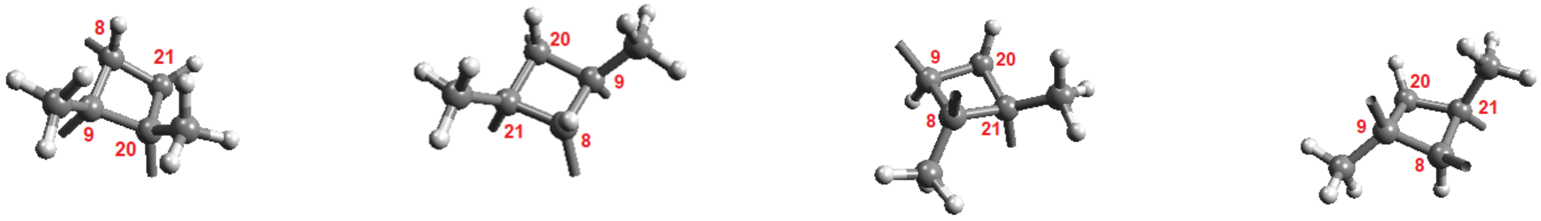

		Cis syn	Cis anti	trans Syn	trans anti
Energy / hartree		-1148.6176720	-1148.62314953	-1148.62285404	-1148.62293274
ΔE / kcal·mol^{-1}		3.3	-0.1	0.1	0.0
Bond lengths / Å	C(8)-C(9)	1.549 (1.546)	1.550 (1.547)	1.549	1.540
	C(20)-C(21)	1.552 (1.542)	1.549 (1.549)	1.549	1.558
	C(8)-C(21)	1.574 (1.571)	1.582 (1.566)	1.602	1.585
	C(9)-C(20)	1.607 (1.596)	1.582 (1.573)	1.567	1.570
Bond angles / °	C(9)-C(20)-C(21)	88.4 (89.0)	89.7 (88.5)	89.7	89.2
	C(8)-C(9)-C(20)	88.5 (89.0)	88.6 (87.3)	89.7	89.0
	C(9)-C(8)-C(21)	89.8 (89.8)	89.7 (88.9)	88.4	89.3
	C(8)-C(21)-C(20)	89.6 (90.0)	88.7 (87.5)	88.4	87.8
Skew angle (degrees) between C(8)-C(9) and C(20)-C(21) bonds		14.9 (11.4)	13.8 (21.3)	15.0	16.6

Table S11 Energies the S_0/S_1 conical intersection and geometrical parameters for the bridging region of dimers, calculated at TDDFT/B3LYP/def2-TZVP and [4,4]CASSCF/def2-SVP (in parentheses) levels of theory. The reported values are averages of S_0 and S_1 energies, with a maximum S_0-S_1 difference of 1.5 kcal/mole for TDDFT and 0.2 kcal/mole for CASSCF. ΔE are differences between the energy of the conical intersection of each isomer and the energy of the trans-anti isomer, taken as reference.

	cis		trans	
	syn	anti	syn	anti

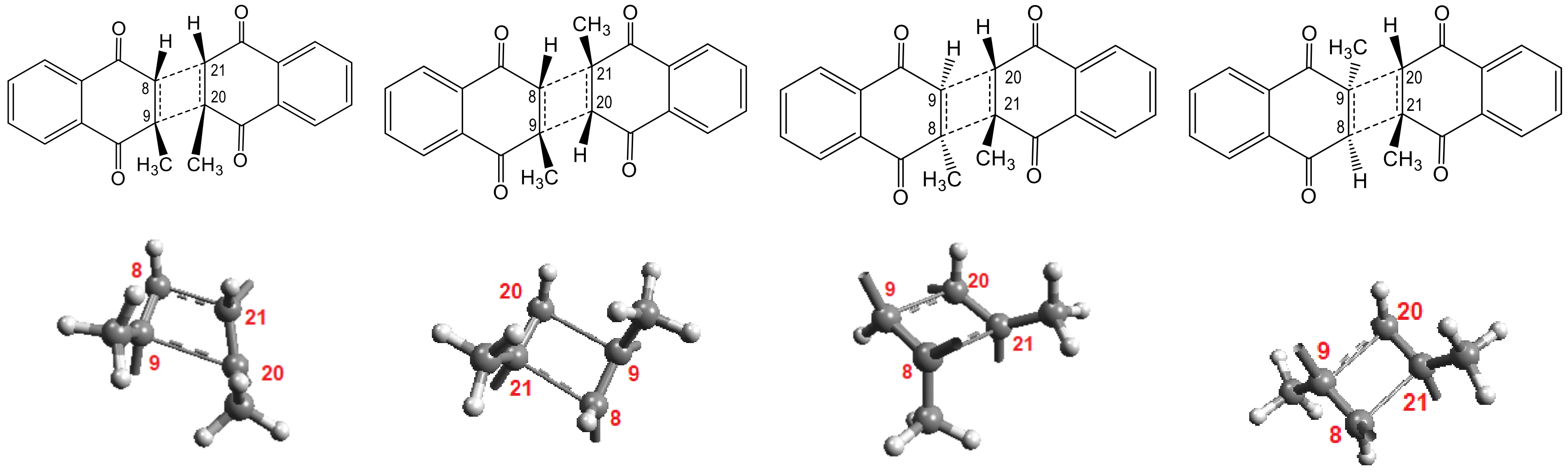

		cis syn	cis anti	trans syn	trans anti
Energy / hartree		-1148.52123009 (-1140.85937417)	-1148.51925793 (-1140.86249613)	-1148.52160193 (-1140.86332444)	-1148.52197609 (-1140.86509648)
ΔE / kcal•mol^{-1}		0.5 (3.6)	1.7 (1.6)	0.2 (1.1)	0.0 (0.0)
Bond lengths / Å	C(8)-C(9)	1.430 (1.454)	1.456 (1.448)	1.450 (1.453)	1.424 (1.446)
	C(20)-C(21)	1.465 (1.456)	1.455 (1.448)	1.437 (1.454)	1.426 (1.447)
	C(8)-C(21)	1.962 (2.160)	2.263 (2.220)	2.427 (2.278)	2.221 (2.224)
	C(9)-C(20)	2.543 (2.294)	2.265 (2.220)	2.058 (2.172)	2.256 (2.233)
Bond angles / °	C(9)-C(20)-C(21)	62.8 (67.3)	83.3 (73.5)	82.9 (72.7)	83.4 (73.9)
	C(8)-C(9)-C(20)	89.4 (103.8)	95.9 (106.5)	111.1 (111.0)	95.2 (106.0)
	C(9)-C(8)-C(21)	83.0 (71.6)	83.3 (73.5)	69.8 (69.2)	84.7 (74.2)
	C(8)-C(21)-C(20)	115.1 (110.3)	96.1 (106.5)	94.3 (105.6)	96.7 (106.0)
Skew angle (degrees) between C(8)-C(9) and C(20)-C(21) bonds		37.4 (24.8)	11.2 (0.4)	19.8 (11.9)	2.0 (5.2)
